# Discovery of 2-Phenylquinolines with Broad-Spectrum Anti-coronavirus Activity

**DOI:** 10.1021/acsmedchemlett.2c00123

**Published:** 2022-05-03

**Authors:** Maria Giulia Nizi, Leentje Persoons, Angela Corona, Tommaso Felicetti, Giada Cernicchi, Serena Massari, Giuseppe Manfroni, Laura Vangeel, Maria Letizia Barreca, Francesca Esposito, Dirk Jochmans, Jessica Milia, Violetta Cecchetti, Dominique Schols, Johan Neyts, Enzo Tramontano, Stefano Sabatini, Steven De Jonghe, Oriana Tabarrini

**Affiliations:** † Department of Pharmaceutical Sciences, University of Perugia, 06123 Perugia, Italy; ‡ Department of Microbiology, Immunology and Transplantation, Laboratory of Virology and Chemotherapy, Rega Institute for Medical Research, KU Leuven, 3000 Leuven, Belgium; § Department of Life and Environmental Sciences, University of Cagliari, Cittadella Universitaria di Monserrato, 09124 Cagliari, Italy

**Keywords:** SARS-CoV-2, 2-Phenylquinolines, Helicase, Repurposing, Autophagy, Pan-CoVs inhibitors

## Abstract

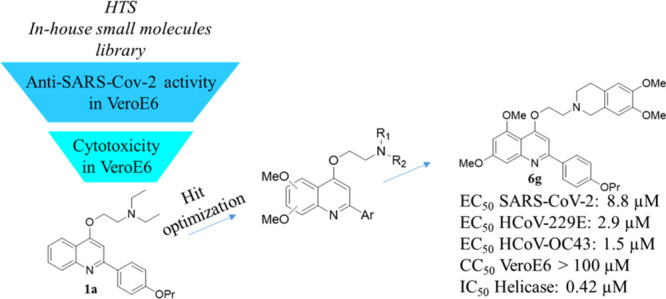

A selection of compounds from a proprietary library, based on chemical diversity and various biological activities, was evaluated as potential inhibitors of the Severe Acute Respiratory Syndrome Coronavirus 2 (SARS-CoV-2) in a phenotypic-based screening assay. A compound based on a 2-phenylquinoline scaffold emerged as the most promising hit, with EC_50_ and CC_50_ values of 6 and 18 μM, respectively. The subsequent selection of additional analogues, along with the synthesis of ad hoc derivatives, led to compounds that maintained low μM activity as inhibitors of SARS-CoV-2 replication and lacked cytotoxicity at 100 μM. In addition, the most promising congeners also show pronounced antiviral activity against the human coronaviruses HCoV-229E and HCoV-OC43, with EC_50_ values ranging from 0.2 to 9.4 μM. The presence of a 6,7-dimethoxytetrahydroisoquinoline group at the C-4 position of the 2-phenylquinoline core gave compound **6g** that showed potent activity against SARS-CoV-2 helicase (nsp13), a highly conserved enzyme, highlighting a potentiality against emerging HCoVs outbreaks.

Currently, the world is in full pandemic crisis due to the Severe Acute Respiratory Syndrome Coronavirus 2 (SARS-CoV-2), the causative agent of COVID-19. Since its first appearance in Wuhan, China, in December 2019, SARS-CoV-2 has rapidly spread around the globe, seriously affecting global health. On March 11, 2020, the World Health Organization declared the SARS-CoV-2 outbreak as a pandemic that had affected an estimated 250 million persons and caused more than 5 million deaths worldwide by January 2022.^1,2^

SARS-CoV-2 belongs to the human coronaviruses (HCoVs), which are a large family of crown-shaped, enveloped, single-stranded, positive-sensed RNA zoonotic viruses that cause various illnesses ranging from a simple cold to a severe acute respiratory syndrome. Other members of the HCoV family include HCoV-229E and HCoV-OC43, causative agents of common cold, and, two highly pathogenic HCoVs, the Middle East Respiratory Syndrome Coronavirus (MERS-CoV) and Severe Acute Respiratory Syndrome Coronavirus 1 (SARS-CoV-1), both responsible for previous epidemics and associated with high morbidity, clearly demonstrating the need for antiviral agents to combat CoV infections. However, the rapid resolution of these epidemics, in combination with a low worldwide spread, led to a global under-investment in CoV drug discovery. As a result, there were no appropriate antiviral drugs available at the time of the COVID-19 outbreak, which now would have paved the way for the control of this pandemic.

The development of vaccines is undoubtedly essential to contain the diffusion of the current virus, and a joint effort never seen before led to a worldwide vaccination campaign in less than 1 year after the SARS-CoV-2 outbreak. The success of this approach in reducing deaths and hospitalization is increasingly evident. However, vaccines may be less effective or even ineffective against emerging variants of SARS-CoV-2. In addition, and most importantly, it is still to be determined how long this vaccine-induced immunity will last, and at least one booster vaccination is essential. Therefore, the development of antiviral drugs targeting SARS-CoV-2 is absolutely necessary. During the lag time needed to produce a new vaccine, antiviral drugs are the only weapon to rapidly control viral infections. Moreover, the development of an antiviral therapy is of paramount importance for unvaccinated people and for the treatment of viral infections caused by emerging SARS-CoV-2 strains that evade the vaccine antibody response. Preferably, new antiviral agents should possess broad-spectrum antiviral activity against various HCoVs, since this will increase the likelihood that these drugs will be active against future outbreaks.

The quickest way to find effective COVID-19 treatment options is the drug repurposing approach, enabling the fast introduction of drugs into clinical settings.^3^ Various molecules, including many antiviral drugs, have been already evaluated in large randomized clinical trials,^4^ with the first two drugs receiving marketing approval. The monophosphoramidate nucleoside prodrug remdesivir, originally developed for Ebola virus infections, was the first drug that received emergency use authorization for the treatment of patients with severe manifestations of COVID-19.^5^ Another viral RNA polymerase inhibitor, molnupiravir, originally developed to treat influenza virus infections, has just received approval for oral use in people who have mild to moderate COVID-19 and at least one risk factor for developing severe illness.^6^

Over the years, our laboratory has worked extensively on the design and synthesis of biologically active compounds, resulting in an in-house library that mainly consists of structurally diverse, small heterocyclic molecules. Notably, this library is rich in agents with proven activity against various viruses such as the human immunodeficiency virus,^7^ the hepatitis C virus,^8^ flaviviruses,^9^ human cytomegalovirus,^10^ and the influenza virus.^11,12^

In this Letter, our efforts toward finding novel anti-SARS-CoV-2 agents, based on a first set of proprietary compounds followed by ad hoc synthesis, are described. In addition, preliminary mode-of-action studies were performed in order to elucidate the molecular target of the identified compounds.

## SARS-CoV-2 Screening—Hit Identification

**Figure 1:**
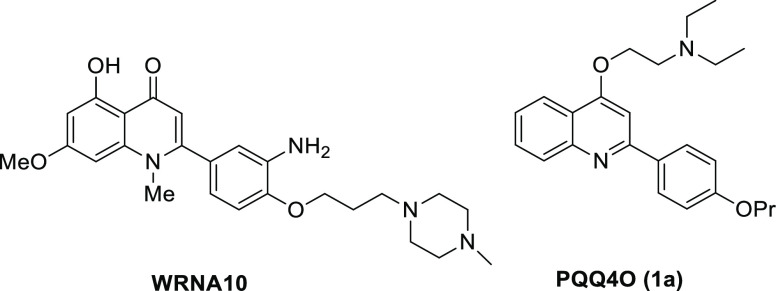
Hits from the primary screening.

In a first round of screening, approximately 100 compounds from our internal library were selected, based on chemical diversity and being endowed with various biological activities (see list of compounds in Table S1). These were evaluated for their antiviral activity against SARS-CoV-2, using SARS-CoV-2-infected VeroE6 cells constitutively expressing an enhanced green fluorescent protein (EGFP), allowing for high-content imaging readout of the virus-induced cytopathic effect.^13^ The cytotoxicity of the compounds in uninfected VeroE6 cells was determined in parallel. GS-441524,^14^ the parent nucleoside analogue of remdesivir, and chloroquine (CQ)^15^ were included as positive controls and reference compounds. From this initial screening, two structurally related hits were identified (Figure 1). The 2-phenylquinolone derivative WRNA10, originally designed as an HIV-1 TAR RNA binder,^16^ showed an EC_50_ value of 10 μM and CC_50_ = 40 μM, while the 2-phenylquinoline analogue PQQ4O^17^ (**1a**) was endowed with EC_50_ and CC_50_ values of 6 μM and 18 μM, respectively.

Compound **1a** belongs to a large series of variously functionalized 2-phenylquinolines (2-PhQs) that have been developed as *S. aureus* NorA efflux pump inhibitors, with compounds that are able to restore very efficiently the ciprofloxacin antibacterial activity coupled with a good safety profile and metabolic stability.^17,18^

Based on the easier synthetic feasibility and availability of more analogues within the proprietary library, the focus was directed toward hit compound **1a**.

## Hit Exploration

The two hits that emerged from this primary screening shared a very similar quinoline core functionalized with a 2-phenyl ring. Of note, only one other compound based on 2-PhQ was initially assayed, the 4-hydroxyquinoline **1**(17) (Table 1), which was completely inactive, highlighting the importance of the basic side chain in imparting antiviral activity. Based on these preliminary insights, a new set of analogues of hit **1a** was selected from the in-house library. In parallel, new congeners were designed and synthesized in order to complete the structure–activity relationship (SAR) study.

**Table 1:**
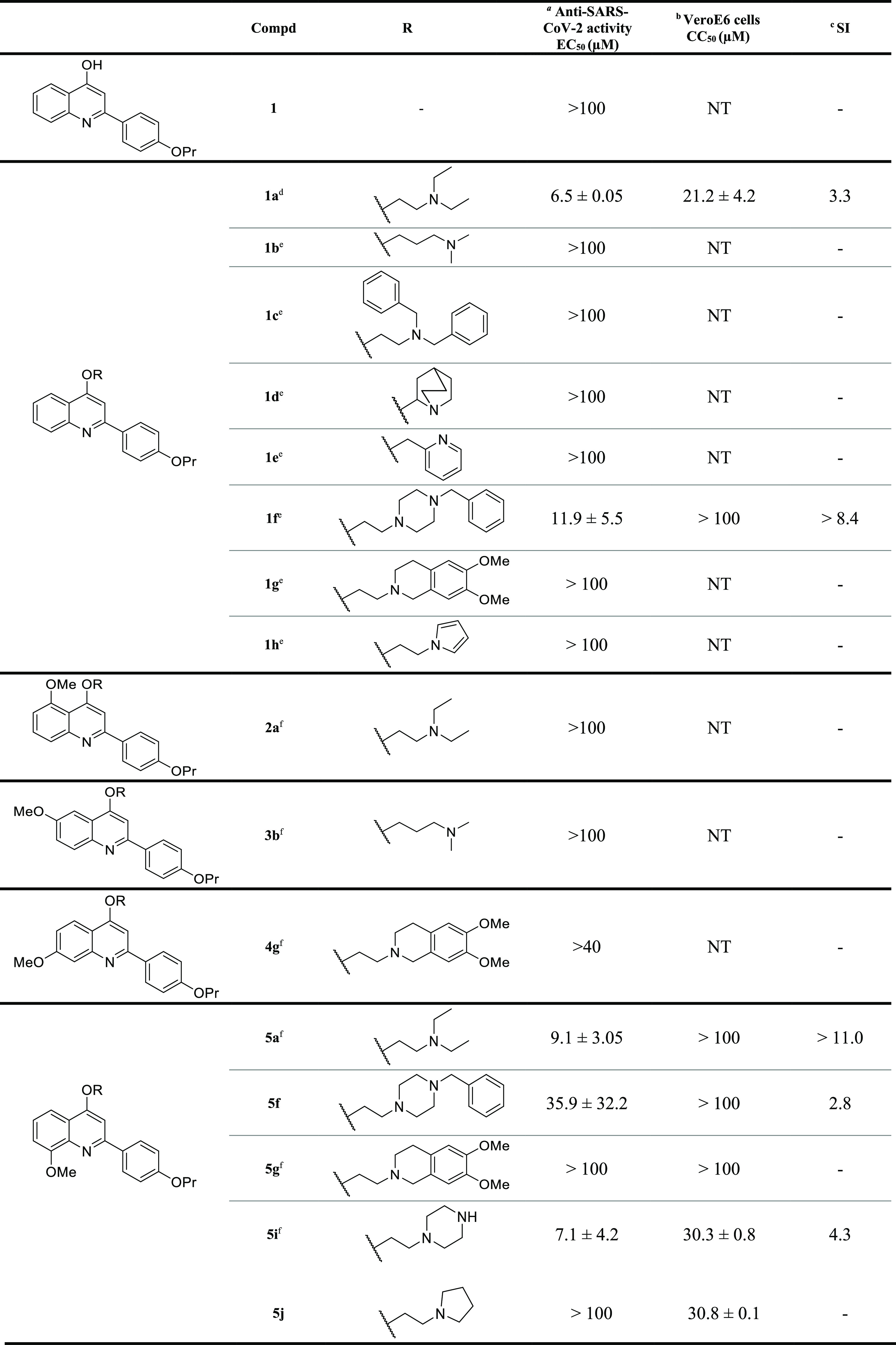

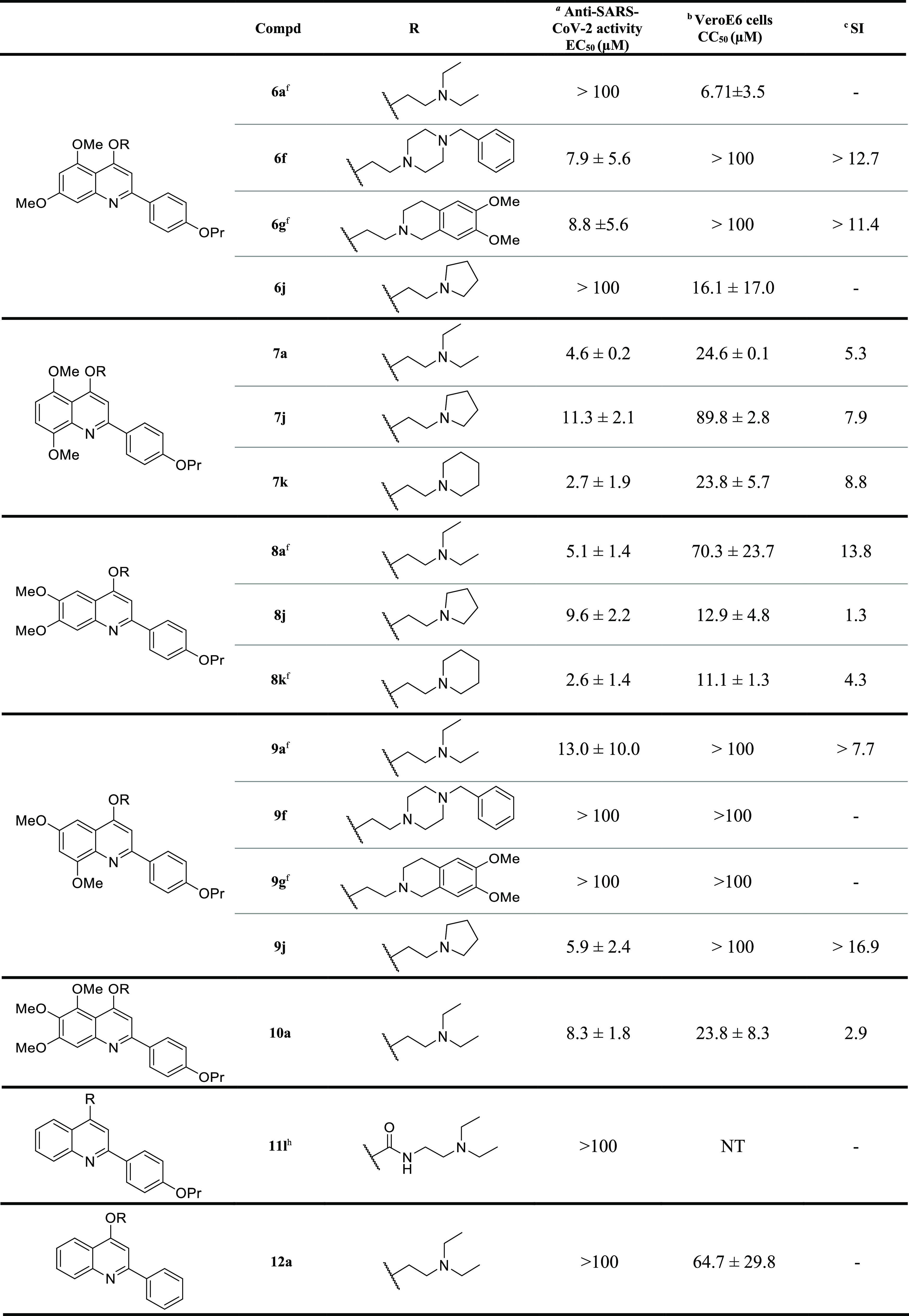

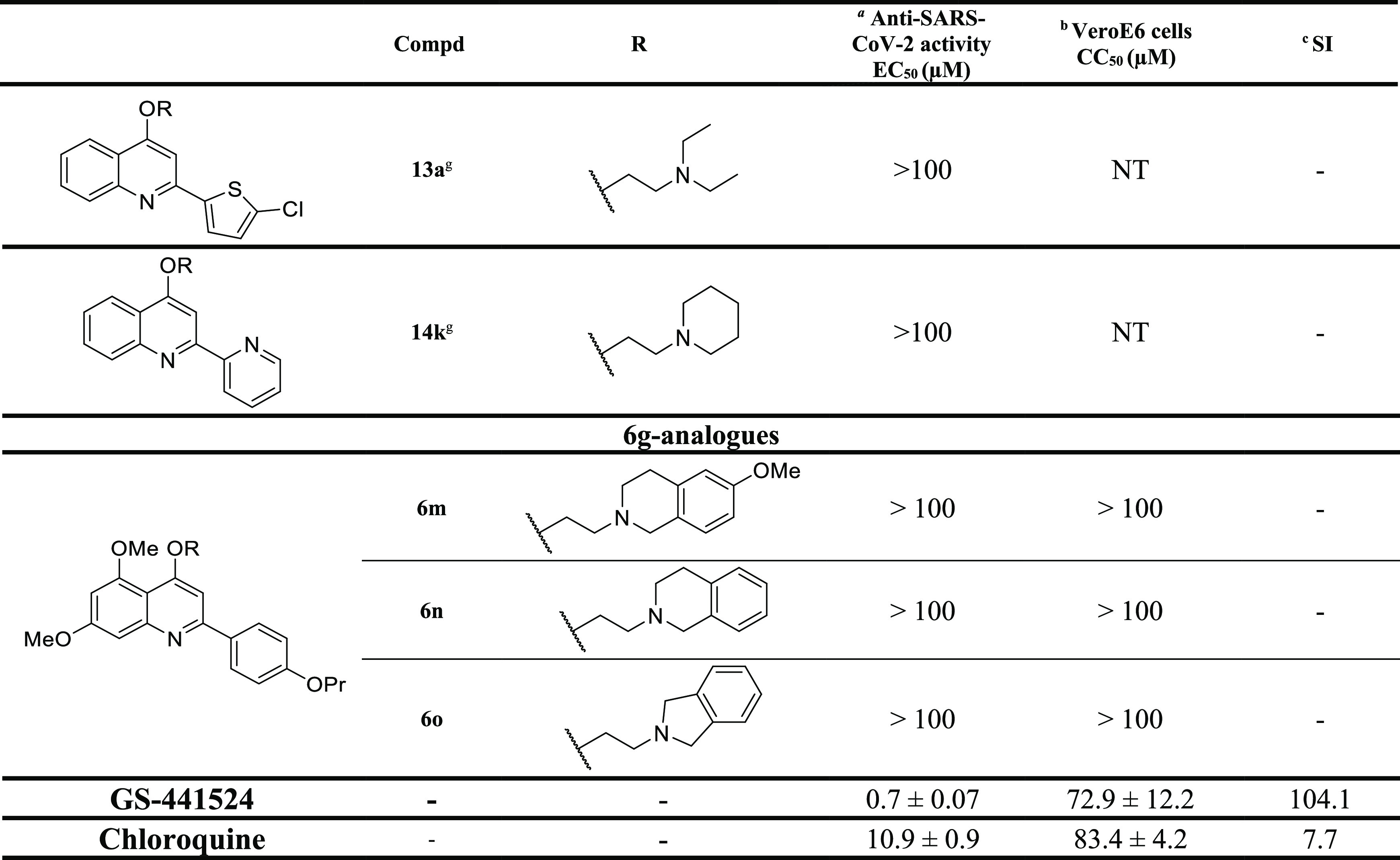
Structure, Anti-SARS-CoV-2 Activity, and Cytotoxicity of 2-PhQs

a EC_50_ = concentration of compound that gives 50% rescue of the virus-reduced eGFP signals as compared to the untreated virus-infected control cells.

b CC_50_ = 50% cytotoxic concentration, as determined by measuring the cell viability with the colorimetric formazan-based MTS assay. The values represent the means ± SD of data derived from duplicate experiments.

c SI = selectivity index calculated as the ratio between CC_50_ and EC_50_ of each compound.

d Ref (17).

e Ref (19).

f Ref (18).

g Ref (20).

h Ref (21).

All the quinolines studied, either available from the library (**1b**–**h**, **2a**, **3b**, **4g**, **5a**,**g**,**i**, **6a**,**g**, **8a**,**k**, **9a**,**g**, **11l**, **13a**, **14k**) or synthesized ad hoc (**5f**,**j**, **6f**,**j**,**m–o, 7a**,**j**,**k**, **8j**, **9f**,**j**, **10a**, **12a**) (Table 1), were decorated at the C-4 position with various moieties with different basicity, such as (cyclo)alkylamines and aromatic amines, but also bulkier substituents such as a dimethoxytetrahydroisoquinoline or a benzylpiperazine. The moieties were linked to the quinoline scaffold via an ether linker, mostly represented by an ethoxy linker, but longer or shorter spacers were also present in a few cases. In the majority of the derivatives, the quinoline core was decorated with one or two methoxy groups at various positions. The presence of a 2-*p*-propoxyphenyl moiety was characteristic for all the tested compounds, with the exception of compounds **12a**, **13a**, and **14k**.

**Scheme 1:**
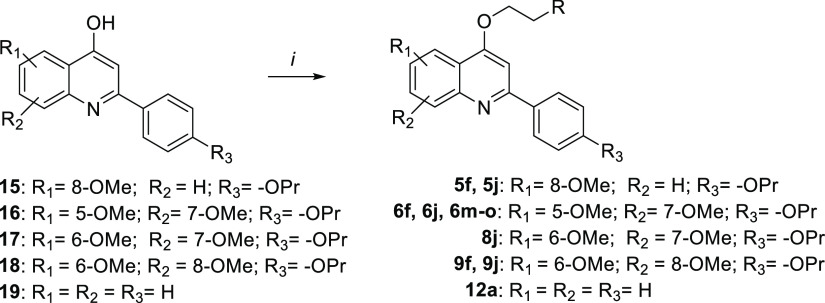
Reagents and conditions: (*i*) 1-(2-chloroethyl)amines (for R, see Table 1), K_2_CO_3_, dry DMF, 80 °C, 4–24 h, 25–51%.

Compounds **5f**,**j**, **6f**,**j**,**m–o**, **8j**, **9f**,**j**, and **12a** (Scheme 1) were prepared by alkylation of mono- and dimethoxy-2-(4-propoxyphenyl)quinolin-4-ol scaffolds **15**–**18** and commercial quinoline **19** with appropriate chloroalkylamines, in moderate yields ranging from 25% to 51%. However, this rather low yield was not due to competitive N-alkylation, since this was prevented by the presence of a bulky phenyl ring at the C-2 position.

**Scheme 2:**
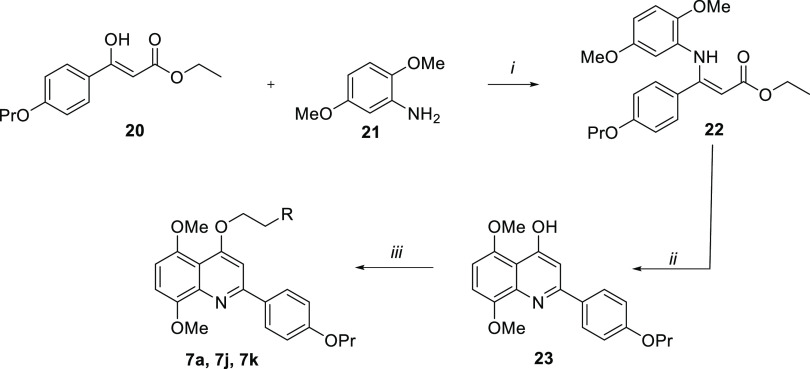
Reagents and conditions: (*i*) *p*-TsOH, dry benzene, reflux, 24 h, 22%; (*ii*) Dowtherm A, 240 °C, 1.5 h, 52%; (*iii*) 1-(2-chloroethyl)-amines (for R, see Table 1), K_2_CO_3_, dry DMF, 80 °C, 4–24 h, 20–38%.

For the synthesis of 5,8-dimethoxy-2-(4-propoxyphenyl)quinoline derivatives **7a**,**j**,**k**, the key intermediate **23** was prepared as shown in Scheme 2. Acrylate **20**(19) was reacted with 2,5-dimethoxyaniline **21** in the presence of catalytic *p*-TsOH in dry benzene, affording aminoacrylate **22** that was heated in a mixture of diphenyl and diphenyl oxide (Dowtherm A) at 240 °C, yielding the 5,8-dimethoxyquinoline **23**. The subsequent reaction with various chloroalkylamines furnished the target compounds **7a**,**j**,**k**.

**Scheme 3:**
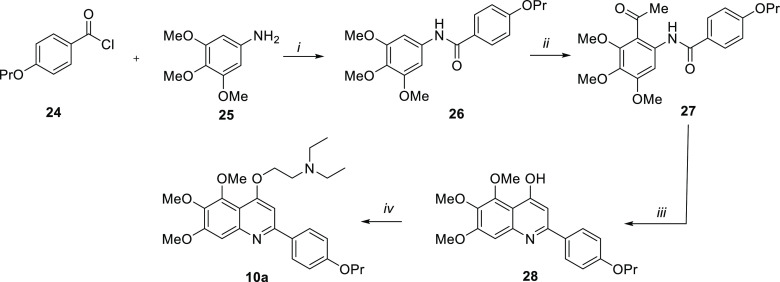
Reagents and conditions: (*i*) Et_3_N, dry THF, 0 °C → rt, 12 h, 98%; (*ii*) SnCl_4_, acetyl chloride, dry CH_2_Cl_2,_ 0 °C → rt, 24 h, 35%; (*iii*) *t*-BuOK, *t*-BuOH, 90 °C, overnight, 68%, (*iv*) 1-(2-chloroethyl)diethylamine, K_2_CO_3_, dry DMF, 80 °C, 3 h, 60%.

The synthesis of the trimethoxy derivative **10a** is depicted in Scheme 3. Intermediate **26**, which was obtained by reacting *p*-propoxybenzoyl chloride **24** with 3,4,5-trimethoxyaniline **25**, was subjected to a Friedel–Crafts acylation with acetyl chloride and SnCl_4_ in dry CH_2_Cl_2_ to give compound **27**. Cyclization of **27** in the presence of *t*-BuOK in *t*-BuOH yielded quinoline **28**, which was alkylated with 1-(2-chloroethyl)diethylamine to give target compound **10a**.

## Antiviral Activity against SARS-CoV-2

Compounds **1**–**14** were tested as inhibitors of SARS-CoV-2 replication, and the data are reported in Table 1. Compounds **9j**, **6f**,**g**, **5a**, **1f**, and **9a** showed antiviral activity, with EC_50_ values ranging from 5.9 to 13.0 μM (Figure S1 reports an example of a microscopic picture of GFP-based phenotypic screening), without any sign of cytotoxicity, even at the highest tested concentration of 100 μM. The highest selectivity index (SI) values were observed for compounds that have a couple of methoxy groups on the quinoline ring, such as compounds **9j** (6,8-dimethoxy) and **6f**,**g** (5,7-dimethoxy). Other derivatives, such as **8k**, **7k**, **7a**, **8a**, **5i**, **8j**, **10a**, and **7j**, also showed good antiviral activity (with EC_50_ values from 2.6 to 11.3 μM), but they displayed a more pronounced cytotoxicity for the VeroE6 cells (CC_50_ = 11.1–89.8 μM).

Among the basic side chains in C-4, the piperidine moiety emerged as the most promising to yield antiviral activity, since compounds **7k** and **8k** were the most potent in the SARS-CoV-2 antiviral assay. On the other hand, the presence of a benzylpiperazine moiety, such as in compounds **1f**, **5f**, **6f**. and **9f**, had a variable effect on the antiviral activity, depending on the substitution pattern on the quinoline scaffold, although all of these analogues lacked cytotoxicity. Any other structural variation of hit compound **1a** was unfruitful. Indeed, a loss of activity was observed when (*i*) the ethoxy spacer was replaced by an amide linker (**11l**); (*ii*) the *p-*propoxy group at the 2-phenyl ring was removed (**12a**); or (*iii*) the *p*-propoxyphenyl ring was replaced by a 5-chlorothienyl (**13a**) or 2-pyridinyl (**14k**) moiety.

Overall, the 2-PhQ scaffold emerged as a privileged structure to impart SARS-CoV-2 inhibitory activity. A number of structural features allow us to tune the antiviral activity as well as the cytotoxicity, such as the *p*-propoxyphenyl moiety at C-2 of the quinoline scaffold, the methoxy groups on the quinoline nucleus, and the *O*-alkyl basic side chain at C-4.

The 2-PhQs with the best profile against SARS-CoV-2 (compounds **1f**, **5a**,**i**, **6f**,**6g**, **7a**,**j**,**k**, **8a**,**k**, and **9a**,**j**) were also tested against other HCoVs to evaluate potential broad-spectrum anti-coronavirus activity. An α-coronavirus (HCoV-229E) and another β-coronavirus (HCoV-OC43), which are both causative agents of a common cold, were selected. The antiviral activity, as well as their cytotoxicity, was measured on HEL 299 cells, along with those of CQ and GS-441524, which were used as positive controls (Table 2). Most analogues were active against HCoV-229E and HCoV-OC43, with EC_50_ values ranging from 0.2 to 9.4 μM (for 229E) and from 0.6 to 7.7 μM (for OC43). In general, the 2-PhQs are more active on these two viruses than on SARS-CoV-2, with the exception of compounds **1f** and **9j**. Compounds **8k**, **5i**, **7j**, and **7a** were more active (EC_50_ = 0.2–0.7 μM) than CQ (EC_50_ = 1.3 μM) and GS-441524 (EC_50_ = 0.9 μM) against 229E, with compound **8k** emerging as the most potent, analogously to what is observed for SARS-CoV-2, even if endowed with some cytotoxicity. Most of the 2-PhQs were more cytotoxic in HEL 299 cells when compared to VeroE6 cells. However, their improved antiviral activity still yields favorable SI values.

**Table 2:** Antiviral Activity against HCoV-229E and HCoV-OC43 and Cytotoxicity on HEL 299 Cells of Selected 2-PhQs

compd	HEL 299 cells CC_50_^a^ (μM)	HCoV-229E EC_50_^b^ (μM)	SI^c^	HCoV-OC43 EC_50_^b^ (μM)	SI^c^
**1f**	>100	61.3 ± 32.2	>1.6	92.3 ± 11.0	>1.2
**5a**	10.4 ± 0.8	1.3 ± 0.9	8.2	0.6 ± 0.1	17.0
**5i**	8.0 ± 1.3	0.3 ± 0.05	25.6	1.7 ± 0.2	4.7
**6g**	26.7 ± 4.1	2.9 ± 0.05	9.1	1.5 ± 0.1	18.0
**6f**	35.6 ± 0.2	2.2 ± 0.7	16.4	1.9 ± 0.2	18.6
**7a**	9.9 ± 0.8	0.7 ± 0.3	13.8	2.0 ± 0.2	5.1
**7j**	8.7 ± 0.8	0.6 ± 0.3	13.4	1.8 ± 0.7	4.7
**7k**	95.3 ± 3.3	9.4 ± 3.6	10.2	7.7 ± 3.3	12.5
**8a**	9.2 ± 0.1	1.8 ± 0.4	5.2	1.9 ± 0.4	4.9
**8k**	12.3 ± 1.1	0.2 ± 0.2	52.4	1.6 ± 0.2	7.9
**9a**	43.5 ± 5.6	3.5 ± 2.7	12.4	2.2 ± 0.5	19.8
**9j**	>100	40.0 ± 20.8	>2.5	22.7 ± 2.8	>4.4
chloroquine	33.6 ± 1.3	1.3 ± 0.1	25.1	<0.8	>40.8
GS-441524	>100	0.9 ± 0.1	>112.4	1.3 ± 0.07	>80.0

a CC_50_ = 50% cytotoxic concentration, as determined by measuring the cell viability with the colorimetric formazan-based MTS assay.

b EC_50_ = Effective concentration producing 50% inhibition of virus-induced cytopathic effect, as determined by measuring the cell viability with the colorimetric formazan-based MTS assay. The values represent the means ± SD of data derived from duplicate experiments.

c SI = Selectivity index calculated as the ratio between CC_50_ and EC_50_.

Overall, the 2-PhQs are a promising compound class with pan-anti-CoV activity.

## Mode of Action Study

To date, no quinoline derivatives functionalized with a phenyl ring at C-2 have been reported as possessing anti-CoV activity.^22^ However, other quinoline analogues endowed with a plethora of pharmacological activities, including antiviral properties,^23^ such as the anti-malarial drugs CQ and hydroxychloroquine, display pronounced in vitro activity against SARS-CoV-1, MERS, and SARS-CoV-2.^24^ Although their clinical use was revoked for safety issues,^25^ more than 90 clinical trials are still in progress to elucidate their therapeutic value.^4^ Among the several hypotheses that have been put forward to explain their antiviral mechanism of action,^26,27^ inhibition of autophagy by impairing autophagosome fusion with lysosomes and thereby stalling the autophagic flux received most attention.^28^

**Figure 2:**
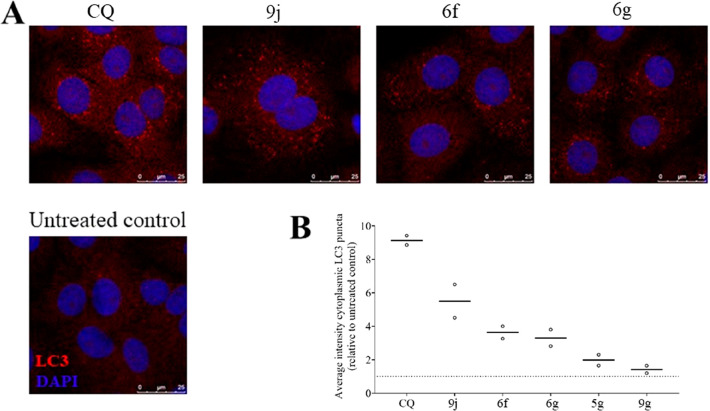
Chloroquine (CQ) treatment (10 μM for 4 h) of VeroE6 cells leads to accumulation of light chain 3-II (LC3-II, red) as visualized by immunofluorescence staining (A, first panel). Treatment with 10 μM of 2-phenylquinolines **9j**, **6f**, and **6g** leads to a similar but less pronounced inhibition of these fusion events. Untreated control cells are shown in the bottom panel; LC3-II localization is shown in red, and nuclei were visualized with DAPI (blue), scale bar 25 μm. High content analysis of the treated cells allows to measure and quantify the increase in cytoplasmic LC3 puncta (B).

Since the 2-PhQs and CQ share the same quinoline scaffold, a selection of 2-PhQs (**5g**, **6f**,**g**, and **9g**,**j**) was evaluated for autophagy inhibition in VeroE6 cells, and compared to CQ (Figure 2). Confocal microscopy was used in which light chain 3-II (LC3-II, a well-known marker of autophagy) was visualized by immunofluorescence staining, whereas the nuclei were visualized by fluorescent staining with 4′,6-diamidino-2-phenylindole (DAPI). Treatment of VeroE6 cells with CQ as positive control led to a 9-fold increase in the intensity of the cytoplasmic LC3 puncta. The tested 2-PhQs demonstrated a less pronounced increased intensity of the cytoplasm of LC3-positive puncta, with compounds **5g** and **9g**, that are devoid of antiviral activity, that showed a minimal effect on the autophagy. Overall, these results show that inhibition of autophagy marginally contributes to the antiviral activity, suggesting that alternative molecular targets might be responsible for the antiviral efficacy of the 2-PhQs.

Recently, many quinoline and quinazoline derivatives have been tested in a cell-based SARS-CoV-2 RdRp reporter system, with a few quinolines being active.^29^ This prompted us to investigate the most potent 2-PhQs (compounds **6f**,**g**, **7k**, and **9j**) as potential SARS-CoV-2 RdRp inhibitors via a fluorescent-based assay, including non-nucleotide inhibitor F243^30^ as a positive control. All the tested compounds lacked activity against RdRp at the highest tested concentration of 30 μM (Table 3).

**Table 3:** Inhibitory Activity of Selected 2-PhQs against SARS-CoV-2 Non-structural Proteins

		helicase	
compd	RdRp^a^ IC_50_ (μM)	unwinding^b^ IC_50_ (μM)	ATPase^c^ IC_50_ (μM)
**6f**	>30 (100%)^d^	>30 (55%)^d^	>30 (96%)^d^
**6g**	>30 (62%)^d^	0.42 ± 0.23	>30 (90%)^d^
**7k**	>30 (100%)^d^	1.41 ± 0.64	>30 (100%)^d^
**9j**	>30 (100%)^d^	>30 (57%)^d^	>30 (100%)^d^
SSYA10-001	ND	0.046 ± 0.015	>3 (90%)^d^
F243	53 ± 3	ND	ND

a Compound concentration required to inhibit the SARS-CoV-2 RdRp-associated activity by 50%.

b Compound concentration required to inhibit the SARS-CoV-2 nsp13 helicase-associated activity by 50%.

c Compound concentration required to inhibit the SARS-CoV-2 nsp13 ATPase-associated activity by 50%.

d Percentage of control measured in the presence of the highest concentration of tested compound.

The same set of compounds was also evaluated as potential inhibitors of another validated drug target, the NTPase/helicase (nsp13), using the triazole-based compound SSYA10-001,^31^ known for its inhibitory activity against helicase of SARS-CoV-1 and MERS-CoV, as reference compound. The SARS-CoV-2 nsp13 helicase uses the energy derived from the hydrolysis of nucleotides to unwind the double-stranded nucleic acids in two single strands along the 5′→3′ direction and possesses two associated activities: RNA unwinding and 5′-triphosphatase (NTPase).^32^

While all the compounds were inactive against the unwinding independent ATPase activity, compounds **6g** and **7k** showed promising activity against the helicase unwinding activity with IC_50_ values of 0.42 and 1.41 μM, respectively. The other analogues showed only moderate nsp13 inhibition, reducing the enzymatic activity by 45% at 30 μM (Table 3).

Thus, the 2-PhQ scaffold seems to have emerged as suitable to inhibit the nsp13 helicase activity, with various potency depending on the substitution patterns around the quinoline ring.

Based on the results achieved on helicase, to better elucidate the role of the dimethoxy tetrahydroisoquinoline of the most potent **6g**, a few analogs were designed by removing one or both of the methoxy groups, as in compounds **6m** and **6n**, or by reducing the size of the bicycle, giving the dihydroisoindole derivative **6o**. All these structure modifications gave compounds devoid of any activity on SARS-CoV-2 replication (Table 1), confirming **6g** as the right combination to impart both antiviral and anti-helicase activity.

In conclusion, screening a proprietary compound library as potential inhibitors of SARS-CoV-2 replication led to the identification of the 2-PhQ **1a**, displaying an EC_50_ value of 6 μM and a low SI of 3. The subsequent testing of close analogues, along with the synthesis of properly designed derivatives, confirmed the 2-PhQ scaffold as being very suitable to impart anti-SARS-CoV-2 activity. The correct functionalization of the quinoline core with two methoxy groups coupled with a C-2 *p*-propoxyphenyl ring and a suitable basic ethoxy side chain at C-4 yielded derivatives showing EC_50_ values ranging from 2.6 to 13 μM and lacking cytotoxicity (CC_50_ > 100 μM). Most analogues were also active against other HCoVs (such as OC43 and 229E) with low μM activity (0.2–9.4 μM), thus emerging as pan-CoV inhibitors with the potential to hit also future HCoV outbreaks.

Preliminary studies on the mechanism of action revealed that 2-PhQs were weak inhibitors of the autophagy pathway and were inactive as RdRp inhibitors; on the other hand, some analogues inhibited the helicase unwinding activity of nsp13 with low μM potency. Nsp13 helicase is a very promising target, being highly conserved, with a 99.8% sequence identity shared between SARS-CoV-2 and SARS-CoV-1, suggesting that drugs targeting nsp13 will be active against emerging HCoVs outbreaks. For compounds **6g** and **7k**, the helicase inhibition appears to mainly contribute to the antiviral activity, with bis-dimethoxyquinoline derivative **6g** emerging as the most promising.

The synthesis of a few analogues of **6g**, compounds **6m**–**o**, gave clear insights on the essential role of the 6,7-dimethoxytetrahydroisoquinoline moiety in conferring antiviral activity through the helicase unwinding inhibition. The co-crystallographic experiments, in progress for **6g**, will establish the importance of this group for nsp13 inhibition and will guide the design of improved 2-PhQ derivatives.

## Supporting Information Available

Table S1, overview of tested compounds; Figure S1, antiviral activity of compound **6g** in the SARS-CoV-2/VeroE6-eGFP assay; experimental details and synthetic procedures; ^1^H NMR, ^13^C NMR, and HPLC for the final compounds; biological experimental procedures (PDF)

## Abbreviations

SARS-CoV-2: Severe Acute Respiratory Syndrome Coronavirus 2
SARS-CoV-1: Severe Acute Respiratory Syndrome Coronavirus 1
MERS-CoV: Middle East Respiratory Syndrome
HCoV: human coronavirus
2-PhQ: 2-phenylquinoline
CQ: chloroquine
LC3-II: light chain 3-II
RdRp: RNA-dependent RNA polymerase
SAR: structure–activity relationship
TLC: thin-layer chromatography
