# Supplementary material for: Discovery of 2-Phenylquinolines with Broad-Spectrum Anti-coronavirus Activity
Source: ACS Med Chem Lett. 2022 May 3;13(5):855–64. doi: 10.1021/acsmedchemlett.2c00123 (PMC9088073; doi:10.1021/acsmedchemlett.2c00123)
Supplement: Supplementary file 1 — ml2c00123_si_001.pdf [file ml2c00123_si_001.pdf]

Supporting Information

Discovery of 2-phenylquinolines with broad-spectrum anti-coronavirus activity

Maria Giulia Nizi,[1] Leentje Persoons,[2] Angela Corona,[3] Tommaso Felicetti,[1] Giada Cernicchi,[1] Serena Massari,[1] Giuseppe Manfroni,[1] Laura Vangeel,[2] Maria Letizia Barreca,[1] Francesca Esposito,[3] Dirk Jochmans,[2] Jessica Milia,[3] Violetta Cecchetti,[1] Dominique Schols,[2] Johan Neyts,[2] Enzo Tramontano,[3] Stefano Sabatini,[1]* Steven De Jonghe,[2]* and Oriana Tabarrini[1]*

[1] Department of Pharmaceutical Sciences, University of Perugia, Perugia, Italy
[2] KU Leuven, Department of Microbiology, Immunology and Transplantation, Laboratory of Virology and Chemotherapy, Rega Institute for Medical Research, Leuven, Belgium
[3] Department of Life and Environmental Sciences, University of Cagliari, Cittadella Universitaria di Monserrato, Cagliari, Italy.

1. **Table S1.** Examples of the main classes of in-house compounds that were tested for anti-SARS-CoV-2 activity on HTS. S2-S3
2. **Figure S1.** Antiviral activity of compound **6g** in the SARS-CoV-2/VeroE6-eGFP assay. S4

EXPERIMENTAL SECTION

3. **Chemistry**

   3.1 Synthetic procedures for the synthesis of all the quinoline derivatives S4-S13

   3.2 ^{1}H-NMR and ^{13}C-NMR spectra of exemplary target compounds. S13-S19

   3.3 HPLC analysis of exemplary target compounds S19-S21

4. **Biology**
   - 4.1 Cells and viruses S22
   - 4.2 SARS-CoV-2 screening S22
   - 4.3 HCoV screening S23
   - 4.4 Autophagy Inhibition assay S23
   - 4.5 Determination of SARS-CoV-2 nsp13 unwinding-associated activity S23
   - 4.6 Determination of SARS-CoV-2 nsp13 ATPase-associated activity S24
   - 4.7 Determination of SARS-CoV-2 nsp12 RdRp activity S24
5. **References** S25-S27

1. Table S1. Examples of the main classes of in-house compounds that were tested for anti-SARS-CoV-2 activity on HTS.

Compd.	**Structure**	**Anti-SARS-CoV-2 activity EC_{50} (μM)** [a]	**VeroE6 cells CC_{50} (μM)** [b]
Anti-HIV/anti-Flu cycloheptathiophenes (32 compounds tested)			
T73[1]	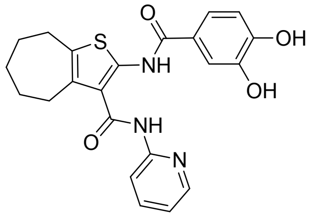	> 100	-
T149[2]	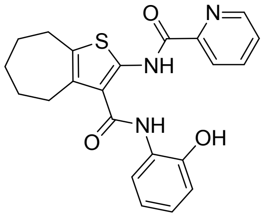	50.70	-
Anti-Flu triazolopyrimidines (17 compounds tested)			
47[3]	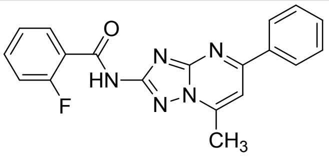	30.61	-
49[3]	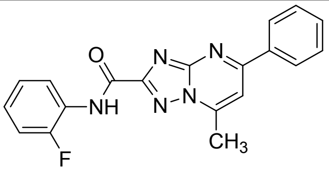	34.47	-
Antitubercular phenothiazines (5 compounds tested)			
MKT-29HCl[4]	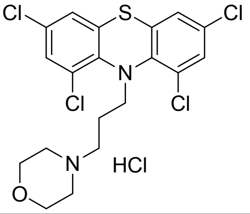	34.8	> 50
PARP inhibitors benzamides (3 compounds tested)			
OUL-35[5]	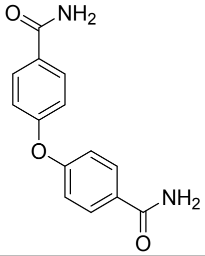	> 100	-
CDK9/TNKS inhibitors (7 compounds tested)			

PTEFbI-19[6]	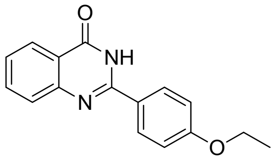	> 100	-
Anticancer/anti-BVDV acridones (2 compounds tested)			
W4Y[7]	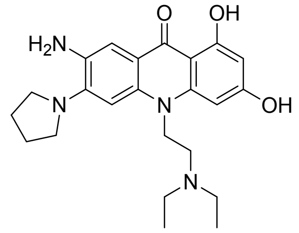	> 100	-
Anti-HIV/antibacterial quinolones (25 compounds tested)			
WM5HCl[8]	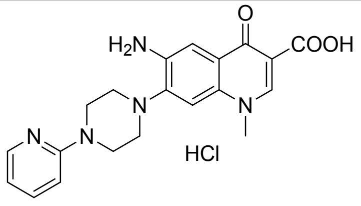	> 100	-
WCF[8]	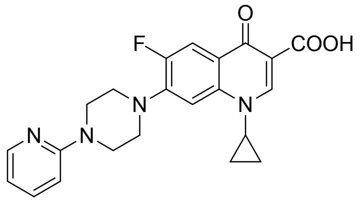	> 100	-
Anti-HIV/HPV naphthyridones (3 compounds tested)			
HP12-13N[9]	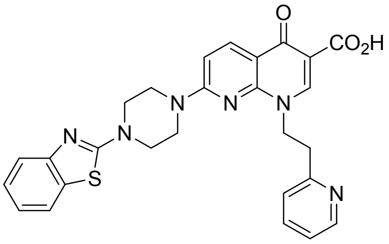	> 100	-
Anti-huntington bisguanidines (3 compounds tested)			
HD04[10]	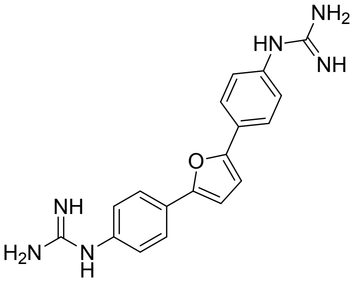	> 100	-
HIV TAR binders (4 compounds tested)			
FQ1[11]	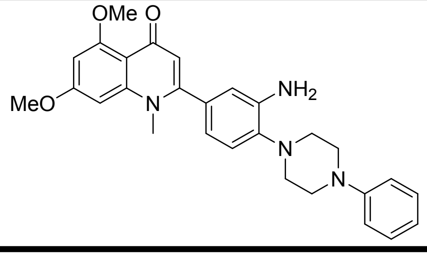	>100	-

[a]EC_{50} = concentration of compound that gives 50% rescue of the virus-reduced eGFP signals as compared to the untreated virus-infected control cells. [b]CC_{50} = 50% cytotoxic concentration, as determined by measuring the cell viability with the colorimetric formazan-based MTS assay.

2. **Figure S1**. Antiviral activity of compound **6g** in the SARS-CoV-2/VeroE6-eGFP assay.

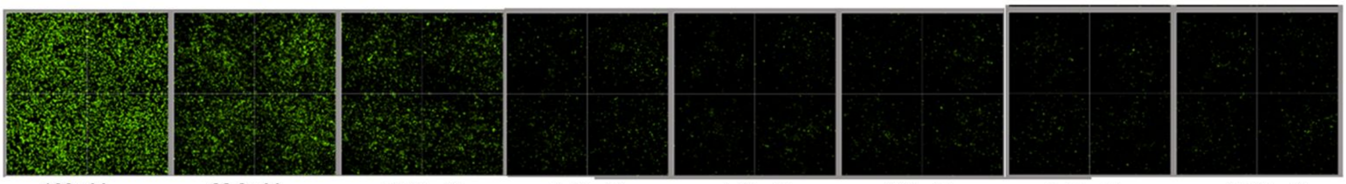

Wells of SARS-CoV2 infected and compound **6g** treated VeroE6-EGFP cells are shown. The numbers indicate the concentration (in μM) of compound **6g**. At higher concentrations (100, 33.3, 11.11, 3.7 μM), a green fluorescence is observed, showing that VeroE6 cells survive SARS-CoV2 infection, demonstrating a clear antiviral effect of compound **6g**.

EXPERIMENTAL SECTION

3. Chemistry

3.1 Synthetic procedures for the synthesis of all the quinoline derivatives

General Chemistry

All starting materials, reagents, and solvents were purchased from common commercial suppliers and were used as such without further purification. Organic solutions were dried over anhydrous Na_2SO_4 and concentrated with rotary evaporator at low pressure. All the reactions were routinely checked by thin-layer chromatography (TLC) on silica gel 60F254 (Merck) and visualized by using UV and iodine. Flash chromatography separations were carried out on Merck silica gel 60 (mesh 230-400). Yields were of purified products and were not optimized. ^{1}H-NMR spectra were recorded at 400 MHz (Bruker Avance DRX-400) or 200 MHz (Bruker Avance DRX-200), while ^{13}C-NMR spectra were recorded at 101 MHz (Bruker Avance DRX-400). Chemical shift are given in ppm (δ) relative to TMS. Spectra were acquired at 298 °K. Data processing was performed with standard Bruker software XwinNMR, and the spectral data are consistent with the assigned structures. Coupling constants (J) are reported in Hz. The purity of the tested compounds was evaluated by HPLC analysis using Jasco LC-4000 instrument equipped with a UV-Visible Diode Array Jasco-MD4015 (Jasco Corporation, Tokyo, Japan), and XTerra MS C18 column, 5 μm x 4.6 mm x 150 mm (Waters Corporation, Massachusetts, USA). Chromatograms were analyzed by ChromNAV2.0 Chromatography Data System software. The purity of the compounds was performed by HPLC at λ

254 nm, at the λ max of each compound and the absolute maximum of absorbance between 200 and 600 nm. The purity is reported in the description of the single compound, and it is well above of 90%, the peak retention time (ret. time) is given in minutes. All the HPLC analysis were performed in 12 min at 1.0 mL/min isocratic: 80% acetonitrile and 20% water with 0.1% diethylamine.

Target compounds **1**, **1a-h**, **2a**, **3d**, **4g**, **5a**, **5g**, **5i**, **6a**, **6g**, **8a**, **8k**, **9a**, **9g**, **11l**, **13a**, **14k** were synthesized according to the procedures already reported by us previously.[12–16]

General procedure A for the *O*-alkylation of the phenylquinoline intermediates for the synthesis of target compounds 5f, 5j, 6f, 6j, 6m-o, 7a, 7j, 7k, 8j, 9f, 9j, and 10a. Preparation of 4-[2-(4-benzylpiperazin-1-yl)ethoxy]-8-methoxy-2-(4-propoxyphenyl)quinoline (5f). Under nitrogen atmosphere, to a suspension of 8-methoxy-2-(4-propoxyphenyl)quinoline **15**[14] (0.28 g, 0.83 mmol) and K_2CO_3 (0.46 g, 3.32 mmol) in dry DMF (10 mL), 4-benzyl-1-(2-chloro-ethyl)-piperazine (0.39 g, 1.66 mmol) was added. The reaction was stirred at 80 °C for 5 h. The mixture was then poured in water and ice and extracted with EtOAc (x3). The organic layers were washed with brine, dried over Na_2SO_4 and evaporated to dryness under reduced pressure. The obtained residue was purified by flash column chromatography (CH_2Cl_2: MeOH 95:5) to give target compound **5f** as a white solid (28% yield, mp 95.0- 97.0 °C). ^{1}H-NMR (400 MHz, $CDCl_3$) δ = 1.09 (3H, t, J = 7.4 Hz, $OCH_2CH_2\mathit{CH_3}$), 1.85-1.90 (2H, m, $OCH_2\mathit{CH_2}CH_3$), 2.54-2.57 (4H, m, piperazine *CH2* x2), 2.73-2.75 (4H, m, piperazine *CH2* x2), 3.04 (2H, t, J = 5.7 Hz, $OCH_2\mathit{CH_2}N$), 3.54 (2H, s, Bn CH_2), 4.02 (2H, t, J = 6.6 Hz , $O\mathit{CH_2}CH_2CH_3$), 4.10 (3H, s, $O\mathit{CH_3}$), 4.43 (2H, t, J = 5.7 Hz, $O\mathit{CH_2}CH_2N$), 7.03 (2H, d, J = 6.9 Hz, H3' and H5'), 7.04-7.08 (1H, m, aromatic H), 7.27-7.29 (2H, m, aromatic H), 7.30-7.34 (4H, aromatic H), 7.34-7.40 (1H, m, aromatic H), 7.73-7.75 (1H, m, aromatic H), 8.12 (2H, d, J = 8.8 Hz, H2' and H6'). ^{13}C-NMR (101 MHz, $CDCl_3$): δ_c 10.44, 22.51, 53.03, 53.70, 56.01, 56.81, 62.99, 66.70, 69.51, 98.46, 108.37, 113.35, 114.50, 121.14, 124.95, 126.99, 128.14, 128.79, 129.15, 132.60, 137.96, 140.99, 155.13, 157.08, 160.18, 161.68. HPLC ret. time = 4.88 minutes; purity (%): 99.86 (254 nm); 97.05 (max abs 200-600); 99.85 (280 nm).

8-Methoxy-2-(4-propoxyphenyl)-4-(2-pyrrolidin-1-ylethoxy)quinoline (5j). The title compound was prepared according to the general procedure A (time = 6 h), starting from intermediate 8-methoxy-2-(4-propoxyphenyl)quinoline **15**[14] (0.25 g, 0.81 mmol) and using 1-(2-chloroethyl)pyrrolidine hydrochloride. After purification by flash column chromatography (CH_2Cl_2: MeOH 95:5), compound **5j** was obtained as a white solid (30% yield, mp 109.0- 111.5 °C). ^{1}H-NMR (400 MHz, $CDCl_3$) δ = 1.09 (3H, t, J = 7.4 Hz, $OCH_2CH_2\mathit{CH_3}$), 1.85-1.90 (6H, m, $OCH_2\mathit{CH_2}CH_3$ and pyrrolidine CH_2 x2), 2.74-2.78 (4H, m, pyrrolidine CH_2 x2), 3.13 (2H, t, J = 5.9 Hz, $OCH_2\mathit{CH_2}N$), 4.02 (2H, t, J = 6.6 Hz, $O\mathit{CH_2}CH_2CH_3$), 4.10 (3H, s, $O\mathit{CH_3}$), 4.45 (2H, t, J = 5.9 Hz, $O\mathit{CH_2}CH_2N$),

7.03 (2H, d, J = 8.9 Hz, H3' and H5'), 7.07 (1H, dd, J = 1.1 and 7.8 Hz, H7), 7.29 (1H, s, H3), 7.39 (1H, t, J = 7.8 Hz, H6), 7.78 (1H, dd, J = 1.2 and 8.4 Hz, H5), 8.12 (2H, d, J = 8.9 Hz, H2' and H6'). ^{13}CNMR (101 MHz, $CDCl_3$): δ_c 10.44, 22.50, 23.53, 54.51, 54.94, 56.02, 67.83, 69.51, 98.45, 108.36, 113.43, 114.50, 121.20, 124.89, 128.79, 132.64, 141.02, 155.14, 157.12, 160.17, 161.77. HPLC ret time = 4.13 minutes; purity (%): 99.68 (254 nm); 97.99 (max abs 200-600 nm); 100 (280 nm).

4-[2-(4-Benzylpiperazin-1-yl)ethoxy]-5,7-dimethoxy-2-(4-propoxyphenyl)quinoline (6f). The title compound was prepared according to the general procedure A (time = 6 h), starting from intermediate 5,7-dimethoxy-2-(4-propoxyphenyl)quinoline **16**[14] (0.09 g, 0.26 mmol) and using 4-benzyl-1-(2-chloro-ethyl)-piperazine. After purification by flash column chromatography ($CHCl_3$: MeOH 95:5), compound **6f** was obtained as a brown oil that was then treated with Et_2O to give a white solid (25% yield, mp 103.5- 105.0 °C). ^{1}H-NMR (400 MHz, $CDCl_3$) δ = 1.09 (3H, t, J = 7.4 Hz, $OCH_2CH_2\mathit{CH_3}$), 1.84-1.90 (2H, m, $OCH_2\mathit{CH_2}CH_3$), 2.53-2.57 (4H, m, piperazine $\mathit{CH_2}$ x2), 2.75-2.79 (4H, m, piperazine $\mathit{CH_2}$ x2), 3.01 (2H, t, J = 5.5 Hz, $OCH_2\mathit{CH_2}N$), 3.56 (2H, s, Bn CH_2), 3.91 (3H, s, $O\mathit{CH_3}$), 3.96 (3H, s, $O\mathit{CH_3}$), 4.02 (2H, t, J = 6.6 Hz, $O\mathit{CH_2}CH_2CH_3$), 4.37 (2H, t, J = 5.5 Hz, $O\mathit{CH_2}CH_2N$), 6.46 (1H, d, J = 2.2 Hz, H6), 7.01-7.04 (3H, m, H3, H3', and H5'), 7.07 (1H, d, J = 2.1 Hz, H8), 7.32 (5H, m, aromatic H), 8.04 (2H, d, J = 8.7 Hz, H2' and H6'). ^{13}C-NMR (101 MHz, $CDCl_3$): δ_c 10.66, 22.69, 53.22, 53.97, 55.63, 56.03, 56.98, 63.20, 67.54, 69.69, 97.61, 98.15, 100.67, 107.31, 114.70, 127.22, 128.35, 128.77, 129.41, 132.35, 137.96, 153.28, 157.89, 158.73, 160.37, 161.03, 163.78. HPLC ret. time = 5.14 minutes; purity (%): 97.22 (254 nm); 96.55 (max abs 200-600 nm); 97.27 (280 nm).

5,7-Dimethoxy-2-(4-propoxyphenyl)-4-(2-pyrrolidin-1-ylethoxy)quinoline (6j). The title compound was prepared according to the general procedure A (time = 12 h), starting from intermediate 5,7-dimethoxy-2-(4-propoxyphenyl)quinoline **16**[14] (0.20 g, 0.59 mmol) and using 1-(2-chloroethyl)pyrrolidine hydrochloride. After purification by flash column chromatography (CH_2Cl_2: MeOH 99:1), compound **6j** was obtained as a white solid (48% yield, mp 84.0- 86.0 °C). ^{1}H-NMR (400 MHz, $CDCl_3$) δ = 1.09 (3H, t, J = 7.3 Hz, $OCH_2CH_2\mathit{CH_3}$), 1.85-1.90 (6H, m, $OCH_2\mathit{CH_2}CH_3$ and pyrrolidine CH_2 x2),2.78-2.80 (4H, m, pyrrolidine CH_2 x2), 3.13 (2H, t, J = 5.7 Hz, $OCH_2\mathit{CH_2}N$), 3.93 (3H, s, $O\mathit{CH_3}$), 3.96 (3H, s, $O\mathit{CH_3}$), 4.02 (2H, t, J = 6.4 Hz, $O\mathit{CH_2}CH_2CH_3$), 4.40 (2H, t, J = 5.9 Hz, $O\mathit{CH_2}CH_2N$), 6.47 (1H, d, J = 2.2 Hz, H8), 7.02-7.04 (3H, m, H3, H3', and H5'), 7.07 (1H, d, J = 2.2 Hz, H6), 8.04 (2H, d, J = 8.7 Hz, H2' and H6'). ^{13}C-NMR (101 MHz, $CDCl_3$): δ_c 10.65, 22.68, 23.70, 54.64, 55.15, 55.63, 56.00, 68.25, 69.69, 97.68, 98.13, 100.69, 107.35, 114.70, 128.77, 132.38, 153.32, 157.92, 158.75, 160.37, 160.99, 163.82. HPLC ret. time = 4.44 minutes; purity (%): 99.62 (254 nm); 96.47 (max abs 200-600 nm); 99.91 (290 nm).

5,7-Dimethoxy-4-[2-(6-methoxy-3,4-dihydroisoquinolin-2(1H)-yl)ethoxy]-2-(4-propoxyphenyl)quinoline (6m). The title compound was prepared according to the general procedure A (time = 3 h), starting from **16**[14] (0.20 g, 0.59 mmol) and using 2-(2-chloroethyl)-6-methoxy-1,2,3,4-tetrahydroisoquinoline. After purification by flash column chromatography (Cyclohexane: EtOAc 50:50), compound **6m** was obtained as a white solid (49% yield, mp 141.0-143.0 °C). ^{1}H-NMR (400 MHz, $CDCl_3$) δ = 1.09 (3H, t, J = 7.4 Hz, $OCH_2CH_2\mathit{CH_3}$), 1.84- 1.89 (2H, m, $OCH_2\mathit{CH_2}CH_3$), 2.96- 3.00 (4H, m, NCH_2), 3.17 (2H, t, J = 5.5 Hz, $OCH_2\mathit{CH_2}N$), 3.80 (3H, s, OCH_3), 3.85 (2H, s, CH_2), 3.95 (3H, s, OCH_3), 3.97 (3H, s, OCH_3), 4.01 (2H, t, J = 6.6 Hz, $O\mathit{CH_2}CH_2CH_3$), 4.46 (3H, t, J = 5.6 Hz, $O\mathit{CH_2}CH_2N$), 6.49 (1H, d, J = 2.3 Hz, H6), 6.68 (1H, d, J = 2.5 Hz, H5”), 6.72 (1H, dd, J = 2.6 and 8.4 Hz, H7”), 6.96 (1H, d, J = 8.4 Hz, H8”), 7.01 (2H, d, J = 6.9 Hz, H3’ and H5’), 7.06 (1H, s, H3), 7.08 (1H, d, J = 2.3 Hz, H8) 8.05 (2H, d, J = 6.8 Hz, H2’ and H6’). ^{13}C-NMR (101 MHz, $CDCl_3$): δ_c 10.64, 22.69, 29.60, 51.79, 55.36, 55.64, 56.07, 56.29, 56.86, 67.96, 69.69,97.72, 98.17, 100.73, 107.38, 112.21, 113.32, 114.71, 127.00, 127.61, 128.77, 132.36, 135.27, 153.32, 157.94, 158.08, 158.75, 160.38, 161.04, 163.84. HPLC: ret. time = 4.76 minutes; purity (%): 99.18 (254 nm), 99.66 (280 nm), 98.74 (max abs 200-600 nm).

4-[2-(3,4-Dihydroisoquinolin-2(1H)-yl)ethoxy]-5,7-dimethoxy-2-(4-propoxyphenyl)quinoline (6n). The title compound was prepared according to the general procedure A (time = 6 h), starting from **16**[14] (0.20 g, 0.59 mmol) and using 2-(2-chloroethyl)-1,2,3,4-tetrahydro-isoquinoline. After purification by flash column chromatography (Cyclohexane: EtOAc 60:40), compound **6n** was obtained as a white solid (39% yield, mp 101.5- 103.0 °C). ^{1}H-NMR (400 MHz, $CDCl_3$) δ = 1.09 (3H, t, J = 7.4 Hz, $OCH_2CH_2\mathit{CH_3}$), 1.84- 1.90 (2H, m, $OCH_2\mathit{CH_2}CH_3$), 2.99- 3.03 (4H, m, NCH_2), 3.19 (2H, t, J = 5.4 Hz, $OCH_2\mathit{CH_2}N$), 3.92 (2H, s, CH_2), 3.97 (3H, s, OCH_3), 3.99 (3H, s, OCH_3), 4.01 (2H, t, J = 6.6 Hz, $O\mathit{CH_2}CH_2CH_3$), 4.47 (2H, t, J = 5.8 Hz, $O\mathit{CH_2}CH_2N$), 6.50 (1H, d, J = 2.1 Hz, H6), 7.03 (2H, d, J = 8.9 Hz, H3’ and H5’), 7.04- 7.08 (3H, m, H3, H8 and aromatic H), 7.12-7.18 (3H, m, aromatic H), 8.04 (2H, d, J = 8.8 Hz, H2’ and H6’). ^{13}C-NMR (101 MHz, $CDCl_3$): δ_c 10.64, 22.68, 29.28, 51.86, 55.64, 56.07, 56.79, 56.86, 67.95, 69.70, 97.71, 98.18, 100.72, 107.37, 114.71, 125.79, 126.33, 126.67, 128.78, 128.84, 132.35, 134.11, 134.76, 153.32, 157.94, 158.76, 160.39, 161.04, 163.83. HPLC: ret. time = 5.09 minutes; purity (%): 97.95 (254 nm); 97.68 (280 nm); 98.34 (max abs 200-600 nm).

4-[2-(1,3-Dihydro-2H-isoindol-2-yl)ethoxy]-5,7-dimethoxy-2-(4-propoxyphenyl)quinoline (6o). The title compound was prepared according to the general procedure A (time = 10 h), starting from **16**[14] (0.280 g, 0.83 mmol) and using 2-(2-chloroethyl)-2,3-dihydro-1H-isoindole. After purification by flash column chromatography ($CHCl_3$: MeOH 99:1), compound **6n** was obtained as a white solid (20% yield, mp 92.5- 94.0 °C). ^{1}H-NMR (400 MHz, $CDCl_3$) δ = 1.09 (3H, t, J = 7.4 Hz,

$OCH_2CH_2\mathit{CH_3}$), 1.84-1.88 (2H, m, $OCH_2\mathit{CH_2}CH_3$), 3.38 (3H, t, *J* = 5.5 Hz, $OCH_2\mathit{CH_2}N$), 3.97 (6H, s, OCH_3), 4.02 (2H, t, *J* = 6.6 Hz, $O\mathit{CH_2}CH_2N$), 4.20 (4H, s, NCH_2), 4.46 (2H, t, *J* = $O\mathit{CH_2}CH_2CH_3$), 6.50 (1H, d, *J* = 2.3 Hz, H6), 7.02 (2H, d, *J* = 8.8 Hz, H3' and H5'), 7.05 (1H, s, H3), 7.09 (1H, d, *J* = 2.2 Hz, H8), 7.22- 7.29 (4H, m, aromatic H), 8.06 (2H, d, *J* = 8.7 Hz, H2' and H6'). ^{13}C-NMR (101 MHz, $CDCl_3$): δ_c 10.64, 22.69, 54.45, 55.64, 55.98, 60.09, 68.78, 69.70, 97.66, 98.14, 100.73, 107.38, 114.72, 122.33, 126.90, 128.78, 132.38, 140.20, 153.36, 157.95, 158.76, 160.39, 161.04, 163.90. HPLC: ret. time= 4.72 minutes; purity (%): 98.45 (254 nm); 98.49 (280 nm); 98.59 (max abs 200-600 nm).

2-{[5,8-Dimethoxy-2-(4-propoxyphenyl)quinolin-4-yl]oxy}-N,N-diethylethanamine (7a). The title compound was prepared according to the general procedure A (time = 4 h), starting from **23** (0.20 g, 0.59 mmol) and using (2-chloroethyl)diethylamine hydrochloride. After purification by flash column chromatography (CH_2Cl_2: MeOH 98:2), compound **7a** was obtained as a yellow solid (25% yield, mp 80.0- 90.0 °C). ^{1}H-NMR (400 MHz, $CDCl_3$) δ = 1.09 (3H, t, *J* = 7.4 Hz, $OCH_2CH_2\mathit{CH_3}$), 1.14 (6H, t, *J* = 7.1 Hz, $NCH_2\mathit{CH_3}$ x2), 1.84-1.90 (2H, m, $OCH_2\mathit{CH_2}CH_3$), 2.77 (4H, q, *J* = 7.1 Hz, $N\mathit{CH_2}CH_3$ x2), 3.12 (2H, t, *J* = 6.0 Hz, $OCH_2\mathit{CH_2}N$), 3.92 (3H, s, OCH_3), 4.02 (2H, t, *J* = 6.6 Hz, $O\mathit{CH_2}CH_2CH_3$), 4.13 (3H, s, OCH_3), 4.34 (2H, t, *J* = 6.3 Hz, $O\mathit{CH_2}CH_2N$), 6.77 (1H, d, *J* = 8.6 Hz, H7), 6.97 (1H, d, *J* = 8.6 Hz, H6), 7.03 (2H, d, *J* = 8.8 Hz, H3' and H5'), 7.29 (1H, s, H3), 8.14 (2H, d, *J* = 8.8 Hz, H2' and H6'). ^{13}C-NMR (101 MHz, $CDCl_3$): δ_c 10.65, 12.03, 22.69, 31.09, 48.014, 51.66, 56.46, 56.80, 69.67, 99.59, 105.59, 108.20, 113.12, 114.64, 128.94, 132.24, 142.93, 149.70, 150.43, 157.25, 160.44, 163.84. HPLC ret. time = 3.89 minutes; purity (%): 93.04 (254 nm); 94.89 (max abs 200-600 nm); 94.33 (284 nm).

5,8-Dimethoxy-2-(4-propoxyphenyl)-4-(2-pyrrolidin-1-ylethoxy)quinoline (7j). The title compound was prepared according to the general procedure A (time = 24 h), starting from **23** (0.15 g, 0.44 mmol) and using 1-(2-chloroethyl)pyrrolidine hydrochloride. After purification by flash column chromatography ($CHCl_3$: MeOH 95:5), compound **7j** was obtained as a white solid (32% yield, mp 127.5- 129.0 °C). ^{1}H-NMR (400 MHz, $CDCl_3$) δ = 1.09 (3H, t, *J* = 5.8 Hz, $OCH_2CH_2\mathit{CH_3}$), 1.84-1.89 (6H, m, $OCH_2\mathit{CH_2}CH_3$ and pyrrolidine CH_2 x2), 2.75-2.79 (4H, m, pyrrolidine CH_2 x2), 3.13 (2H, t, *J* = 6.0 Hz, $OCH_2\mathit{CH_2}N$), 3.92 (3H, s, OCH_3), 4.02 (2H, t, *J* = 6.6 Hz, $O\mathit{CH_2}CH_2CH_3$), 4.06 (3H, s, OCH_3), 4.41 (2H, t, *J* = 6.0 Hz, $O\mathit{CH_2}CH_2N$), 6.77 (1H, d, *J* = 8.7 Hz, H6), 6.97 (1H, d, *J* = 8.7 Hz, H7), 7.03 (2H, d, *J* = 8.9 Hz, H3' and H5'), 7.29 (1H, s, H3), 8.13 (2H, d, *J* = 8.5 Hz, H2' and H6'). ^{13}C-NMR (101 MHz, $CDCl_3$): δ_c 10.43, 22.50, 23.53, 54.46, 54.91, 56.32, 56.73, 68.23, 69.50, 99.42, 105.67, 108.15, 113.07, 114.49, 128.74, 132.14, 141.13, 149.60, 150.31, 157.07, 160.26, 163.62. HPLC ret. time = 3.62 minutes; purity (%): 96.09 (254 nm); 95.98 (max abs 200-600 nm); 98.68 (288 nm).

5,8-Dimethoxy-4-(2-piperidin-1-ylethoxy)-2-(4-propoxyphenyl)quinolone (7k). The title compound was prepared according to the general procedure A (time = 4 h), starting from **23** (0.20 g, 0.59 mmol) and using 1-(2-chloroethyl)piperidine hydrochloride. After purification by flash column chromatography (CH_2Cl_2: MeOH 98:2), compound **7k** was obtained as a white solid (38% yield, mp 120.0- 122.5 °C). ^{1}H-NMR (400 MHz, $CDCl_3$) δ = 1.08 (3H, t, J = 7.4 Hz, $OCH_2CH_2\mathit{CH_3}$), 1.49-1.51 (2H, m, piperidine CH_2), 1.64-1.69 (4H, m, piperidine CH_2 x2), 1.83-1.89 (2H, m, $OCH_2\mathit{CH_2}CH_3$), 2.64-2.68 (4H, m, piperidine CH_2 x2), 3.01 (2H, t, J = 5.8 Hz, $OCH_2\mathit{CH_2}N$), 3.92 (3H, s, OCH_3), 4.01 (2H, t, J = 6.6 Hz, $O\mathit{CH_2}CH_2CH_3$), 4.05 (3H, s, OCH_3), 4.40 (2H, t, J = 5.8 Hz, $O\mathit{CH_2}CH_2N$), 6.77 (1H, d, J = 8.6 Hz, H7), 6.97 (1H, d, J = 8.7 Hz, H6), 7.03 (2H, d, J = 8.8 Hz, H3' and H5'), 7.23 (1H, s, H3), 8.13 (2H, d, J = 8.8 Hz, H2' and H6'). ^{13}C-NMR (101 MHz, $CDCl_3$): δ_c 10.65, 22.69, 24.20, 26.13, 55.36, 56.46, 56.94, 57.70, 67.35, 69.66, 99.55, 105.82, 108.20, 113.20, 114.64, 128.93, 132.25, 142.91, 149.73, 150.43, 157.25, 160.43, 163.75. HPLC ret. time = 4.27 minutes; purity (%): 96.11 (254 nm); 96.66 (max abs 200-600 nm); 98.77 (284 nm).

6,7-Dimethoxy-2-(4-propoxyphenyl)-4-(2-pyrrolidin-1-ylethoxy)quinoline (8j). The title compound was prepared according to the general procedure A (time = 6 h), starting from intermediate 6,7-dimethoxy-2-(4-propoxyphenyl)quinoline **17**[14] (0.25 g, 0.74 mmol) and using 1-(2-chloroethyl)pyrrolidine hydrochloride. After purification by flash column chromatography (CH_2Cl_2: MeOH 97:3), compound **8j** was obtained as a white solid (43% yield, mp 107.0- 109.0 °C). ^{1}H-NMR (400 MHz, $CDCl_3$) δ = 1.09 (3H, t, J = 7.4 Hz, $OCH_2CH_2\mathit{CH_3}$), 1.83-1.90 (6H, m, $OCH_2\mathit{CH_2}CH_3$ and pyrrolidine CH_2 x2), 2.74-2.76 (4H, m, pyrrolidine CH_2 x 2), 3.12 (2H, t, J = 6.0 Hz, $OCH_2\mathit{CH_2}N$), 3.99-4.06 (8H, m, $O\mathit{CH_2}CH_2CH_3$ and OCH_3 x2), 4.45 (2H, t, J = 6.0 Hz, $O\mathit{CH_2}CH_2N$), 7.04 (2H, d, J = 8.8 Hz, H3' and H5'), 7.09 (1H, s, H3), 7.43 (1H, s, H5), 7.45 (1H, s, H8), 8.03 (2H, d, J = 8.8 Hz, H2' and H6'). ^{13}C-NMR (101 MHz, $CDCl_3$): δ_c 10.48, 22.51, 23.51, 54.56, 54.98, 55.90, 56.04, 67.71, 69.50, 97.18, 99.78, 108.05, 114.45, 114.54, 128.40, 132.80, 146.10, 148.45, 152.35, 156.62, 159.88, 160.66. HPLC ret. time = 3.60 minutes; purity (%): 98.92 (254 nm); 95.19 (max abs 200-600 nm); 98.69 (272 nm).

4-[2-(4-Benzylpiperazin-1-yl)ethoxy]-6,8-dimethoxy-2-(4-propoxyphenyl)quinoline (9f). The title compound was prepared according to the general procedure A (time = 4 h), starting from intermediate 6,8-dimethoxy-2-(4-propoxyphenyl)quinoline **18**[14] (0.12 g, 0.35 mmol) and using 4-benzyl-1-(2-chloro-ethyl)-piperazine. After purification by flash column chromatography ($CHCl_3$:MeOH 95:5), compound **9f** was obtained as a white solid (25% yield, mp 104.0- 106.5 °C). ^{1}H-NMR (400 MHz, $CDCl_3$) δ = 1.09 (3H, t, J = 7.4 Hz, $OCH_2CH_2\mathit{CH_3}$), 1.84-1.89 (2H, m, $OCH_2\mathit{CH_2}CH_3$), 2.54-2.58 (4H, m, piperazine $\mathit{CH_2}$ x2), 2.73-2.77 (4H, m, piperazine $\mathit{CH_2}$ x2), 3.05 (2H, t, J = 5.8 Hz, $OCH_2\mathit{CH_2}N$), 3.55 (2H, s, aromatic CH_2), 3.94 (3H, s, OCH_3), 4.01 (2H, t, J = 6.6

Hz , O$CH_2$$CH_2CH_3$), 4.06 (3H, s, OCH_3), 4.43 (2H, t, J = 5.9 Hz, O$CH_2$$CH_2$N), 6.73 (1H, d, J = 2.6 Hz, H5), 7.01 (3H, m, H7, H3' and H5'), 7.20 (1H, s, H3), 7.28-7.35 (5H, m, aromatic H), 8.08 (2H, d, J = 8.9 Hz, H2' and H6'). ^{13}C-NMR (101 MHz, $CDCl_3$): δ_c 10.45, 22.52, 53.02, 53.68, 55.38, 56.07, 56.78, 62.99, 66.59, 69.49, 91.27, 98.93, 101.37, 114.48, 121.32, 127.02, 128.16, 128.48, 129.16, 132.66, 137.44, 137.85, 154.76, 156.22, 157.24, 159.90, 160.75. HPLC ret. time = 4.63 minutes; purity (%): 98.93 (254 nm); 95.69 (max abs 200-600 nm); 99.27 (276 nm).

6,8-Dimethoxy-2-(4-propoxyphenyl)-4-(2-pyrrolidin-1-ylethoxy)quinoline (9j). The title compound was prepared according to the general procedure A (time = 3 h), starting from intermediate 6,8-dimethoxy-2-(4-propoxyphenyl)quinoline **18**[14] (0.23 g, 0.68 mmol) and using 1-(2-chloroethyl)pyrrolidine hydrochloride. After purification by flash column chromatography (CH_2Cl_2: MeOH 95:5), compound **9j** was obtained as a white solid (51% yield, mp 120.5- 120.0 °C). ^{1}H-NMR (400 MHz, $CDCl_3$) δ = 1.09 (3H, t, J = 7.3 Hz, $OCH_2CH_2$$CH_3$), 1.82-1.89 (6H, m, $OCH_2$$CH_2$$CH_3$ and pyrrolidine CH_2 x2), 2.75-2.76 (4H, m, pyrrolidine CH_2 x2), 3.13 (2H, t, J = 5.8 Hz, $OCH_2$$CH_2$N), 3.96 (3H, s, OCH_3), 4.01 (2H, t, J = 6.4 Hz, O$CH_2$$CH_2CH_3$), 4.07 (3H, s, OCH_3), 4.45 (2H, t, J = 5.9 Hz, O$CH_2$$CH_2$N), 6.74 (s, 1H, H7), 7.02 (2H, d, J = 8.7 Hz, H3' and H5'), 7.05 (1H, s, H5), 7.21 (1H, s, H3), 8.08 (2H, d, J = 8.2 Hz, H2' and H6'). ^{13}C-NMR (101 MHz, $CDCl_3$): δ_c 10.65, 22.70, 23.69, 54.69, 55.13, 55.56, 56.26, 67.91, 69.66, 91.53, 99.08, 101.49, 114.64, 121.54, 128.67, 132.84, 137.61, 154.97, 156.37, 157.36, 160.05, 161.00. HPLC ret. time = 3.88 minutes; purity (%): 99.75 (254 nm); 96.06 (max abs 200-600 nm); 99.49 (276 nm).

***N,N*-diethyl-2-{[5,6,7-trimethoxy-2-(4-propoxyphenyl)quinolin-4-yl]oxy}ethanamine (10a).** The title compound was prepared according to the general procedure A (time = 3 h), starting from intermediate **28** (0.30 g, 0.81 mmol) and using (2-chloroethyl)diethylamine hydrochloride. After purification by flash column chromatography (CH_2Cl_2:MeOH 98:2), compound **10a** was obtained as a yellow solid (60% yield, mp 85.0- 87.0 °C).^{1}H-NMR (400 MHz, $CDCl_3$) δ = 1.07-1.16 (9H, m, $OCH_2CH_2$$CH_3$ and $NCH_2$$CH_3$ x2), 1.85-1.90 (2H, m, $OCH_2$$CH_2$$CH_3$), 2.73 (4H, q, J = 7.1 Hz, N$CH_2$$CH_3$ x2), 3.11 (2H, t, J = 6.0 Hz, $OCH_2$$CH_2$N), 3.97 (3H, s, OCH_3), 3.99 (3H, s, OCH_3), 4.02-4.04 (5H, m, OCH_3 and O$CH_2$$CH_2CH_3$), 4.33 (2H, t, J = 6.9 Hz, O$CH_2$$CH_2$N), 7.03 (2H, d, J = 8.7 Hz, H3' and H5'), 7.09 (1H, s, H3), 7.32 (1H, s, H8), 8.04 (2H, d, J = 8.6 Hz, H2' and H6'). ^{13}C-NMR (101 MHz, $CDCl_3$): δ_c 10.63, 12.06, 22.69, 48.02, 51.94, 56.09, 61.47, 62.12, 67.28, 69.72, 97.94, 105.46, 110.35, 114.76, 128.66, 132.46, 141.92, 148.34, 148.76, 155.58, 157.75, 161.32, 162.66. HPLC ret. time = 4.47 minutes; purity (%): 98.47 (254 nm); 96.19 (max abs 200-600 nm); 98.64 (276 nm).

***N,N*-diethyl-2-[(2-phenylquinolin-4-yl)oxy]ethanamine (12a).** The title compound was prepared according to the general procedure A (time = 2 h), starting from 2-phenylquinoline **19** (0.30 g, 1.34

mmol) and using (2-chloroethyl)diethylamine hydrochloride. After purification by flash column chromatography (CH_2Cl_2: aceton 60:40), compound **12a** was obtained as a yellow solid (35% yield, mp 110.0- 112.0 °C). ^{1}H-NMR (200 MHz, $CDCl_3$) δ = 1.18 (6H, t, J = 7.1 Hz, NCH_2CH_3 x2), 2.75 (4H, q, J = 7.2 Hz, NCH_2CH_3 x2), 3.13 (2H, t, J = 6.1 Hz, OCH_2CH_2N), 4.40 (2H, t, J = 6.1 Hz, OCH_2CH_2N), 7.24 (1H, s, H3), 7.46-7.61 (5H, m, aromatic H), 7.71-7.79 (1H, m, aromatic H), 8.11-8.17 (3H, m, aromatic H), 8.21-8.26 (1H, m, aromatic H). ^{13}C-NMR (101 MHz, DMSO): δ_c 9.07, 47.35, 49.55, 65.76, 102.69, 119.88, 123.70, 125.94 128.63, 129.69, 129.89, 132.86, 134.41, 137.92, 145.02, 153.34, 156.97 HPLC ret. time = 3.67 minutes; purity (%): 97.85 (254 nm); 95.79 (max abs 200-600 nm); 96.54 (256 nm).

Ethyl-3-[(2,5-dimethoxyphenyl)amino]-3-(4-propoxyphenyl)acrylate (22). To a solution of **22**[13] (4.0 g, 16 mmol) in dry benzene (150 mL), 2,5-dimethoxy aniline **21** (12.25 g, 80 mmol) and *p*-TsOH (0.80 g, 20% w/w) were added. The reaction mixture was stirred at reflux using Dean-Stark apparatus for 24 h. Then the reaction mixture was concentrated under vacuum, poured in 2 N HCl solution and extracted with CH_2Cl_2 (x3). The organic layers were washed with brine, dried over Na_2SO_4 and evaporated to dryness under reduced pressure. The crude product was purified by flash column chromatography (Pet: Et_2O 80:20) obtaining **22** (22% yield). ^{1}H-NMR (200 MHz, $CDCl_3$) δ = 1.07 (3H, t, J = 7.3 Hz, $OCH_2CH_2CH_3$), 1.35 (3H, t, J = 7.1 Hz, OCH_2CH_3), 1.82-1.86 (2H, m, $OCH_2CH_2CH_3$), 3.42 (3H, s, OCH_3), 3.91 (3H, s, OCH_3), 3.92-3.98 (2H, m, $OCH_2CH_2CH_3$), 4.20-4.31 (2H, q, , J = 7.1 Hz, OCH_2CH_3), 5.02 (1H, s, CH), 5.90 (1H, d, J = 2.9 Hz, H6'), 6.40 (1H, dd, J = 2.9 and 8.9 Hz, H4'), 6.78 (1H, d, J = 8.9 Hz, H3'), 6.87 (2H, d, J = 8.9 Hz, H3 and H5), 7.35 (2H, d, J = 8.9 Hz, H2 and H6), 10.26 (1H, bs, NH).

5,8-Dimethoxy-2-(4-propoxyphenyl)quinolin-4-ol (23). Intermediate **22** (1.20 g, 31 mmol) reacted in Dowtherm A (6 mL) at 240 °C for 1.5 h. After cooling at rt, the mixture was treated with cyclohexane, obtaining **22** as a solid recovered by filtration (52 % yield). ^{1}H-NMR (200 MHz, $CDCl_3$) δ = 1.10 (3H, t, J = 7.4 Hz, OCH_2CH_3), 1.84-1.94 (2H, m, $OCH_2CH_2CH_3$), 3.99-4.03 (8H, m, OCH_3 x2 and $OCH_2CH_2CH_3$), 6.55-6.57 (1H, m, aromatic H), 6.66-6.68 (1H, m, aromatic H), 6.98-6.99 (1H, m, aromatic H), 7.05 (2H, d, J = 8.0 Hz, H3' and H5'), 7.65 (2H, d, J = 8.4 Hz, H2' and H6'), 8.99 (1H, bs, OH).

4-Propoxy-*N*-(3,4,5-trimethoxyphenyl)benzamide (26). Under nitrogen atmosphere, to a suspension of 3,4,5-trimethoxyaniline **25** (3.22 g, 17.60 mmol) in dry THF (20 mL), Et_3N (3.34 g, 33 mmol) was slowly added in 15 minutes at 0 °C. To the mixture, a solution 4-propoxybenzoil chloride **24** (2.18 g, 11 mmol) in dry THF (6 mL) was then added drop wise. The reaction was stirred at r.t for 12 h. The mixture was concentrated under vacuum and then poured in water and ice to give a precipitate that was filtrated, obtaining **26** (98% yield). ^{1}H-NMR (200 MHz, $CDCl_3$) δ = 1.10 (3H, t,

J = 7.4 Hz, $OCH_2CH_2\mathit{CH_3}$), 1.85-1.87 (2H, m, $OCH_2\mathit{CH_2}CH_3$), 3.86-3.90 (9H, m, OCH_3 x3), 4.02 (2H, t, J = 6.6 Hz, $O\mathit{CH_2}CH_2CH_3$), 6.98-7.02 (4H, m, H3, H5, H2', and H6'), 7.80 (1H, bs, NH), 7.87 (2H, d, J = 8.8 Hz, H2' and H6').

***N*-(2-acetyl-3,4,5-trimethoxyphenyl)-4-propoxybenzamide (27).** Under nitrogen atmosphere, to a solution of **26** (3.47 g, 10 mmol), in dry CH_2Cl_2 (50 mL) $SnCl_4$ (10.42 g, 40 mmol) and a solution of acetyl chloride (3.13 g, 40 mmol) in dry CH_2Cl_2 (5 mL) were added at 0 °C. The reaction mixture was stirred at r.t. for 24 h, then poured in 2 N HCl solution and extracted with EtOAc (x3). The organic layers were washed with brine, dried over Na_2SO_4 and evaporated to dryness under reduced pressure. The crude product was purified by flash column chromatography (Pet: EtOAc 90:10) obtaining **27** as a solid (35% yield). ^{1}H-NMR (200 MHz, $CDCl_3$) δ = 1.10 (3H, t, J = 7.4 Hz, $OCH_2CH_2\mathit{CH_3}$), 1.87-1.91 (2H, m, $OCH_2\mathit{CH_2}CH_3$), 2.71 (3H, s, CH_3), 3.89 (3H, s, OCH_3), 4.00- 4.07 (8H, m, $O\mathit{CH_2}CH_2CH_3$ and OCH_3 x 2), 7.03 (2H, d, J = 8.9 Hz, H3 and H5), 8.02 (2H, d, J = 8.9 Hz, H2 and H6), 8.46 (1H, s, H6').

5,6,7-Trimethoxy-2-(4-propoxyphenyl)quinolin-4-ol (28). To a solution of intermediate **27** (0.55 g, 1.54 mmol) in *t*-BuOH (10 mL) *t*-BuOK (0.86 g, 7.72 mmol) was added. The reaction mixture was stirred at 90 °C overnight and then concentrated under vacuum and poured in a 2 N HCl solution. Intermediate **28** obtained as a solid was filtrated (68 % yield). ^{1}H-NMR (200 MHz, $CDCl_3$) δ = 1.09 (3H, t, J = 7.4 Hz, $OCH_2CH_2\mathit{CH_3}$), 1.83-1.90 (2H, m, $OCH_2\mathit{CH_2}CH_3$), 4.02-4.09 (5H, m, $O\mathit{CH_2}CH_2CH_3$ and $O\mathit{CH_3}$), 4.19 (3H, s, $O\mathit{CH_3}$), 4.34 (3H, s, $O\mathit{CH_3}$), 7.09-7.16 (3H, m, H8, H3' and H5'), 8.29 (2H, d, J = 9.0 Hz, H2' and H6'), 8.91 (1H, bs, OH).

3.2 ^{1}H-NMR and ^{13}C-NMR exemplary spectra of target compounds

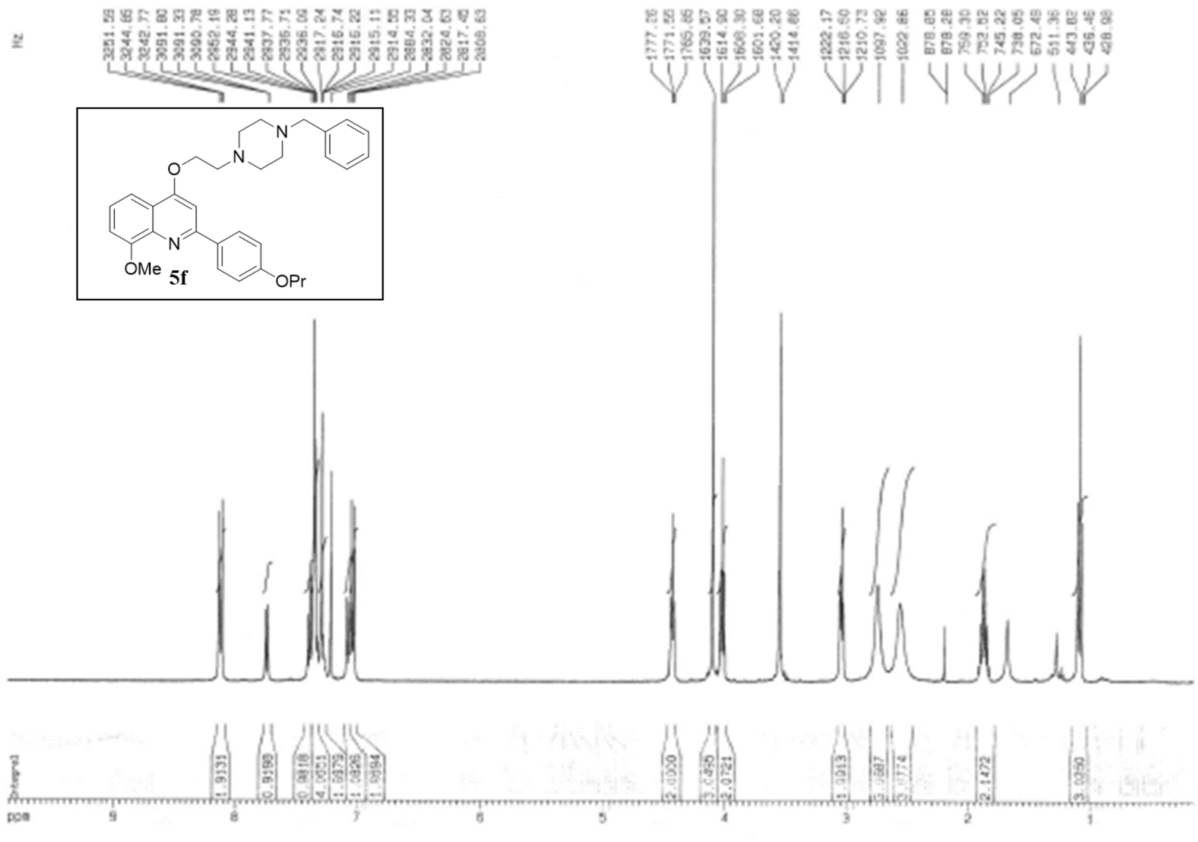

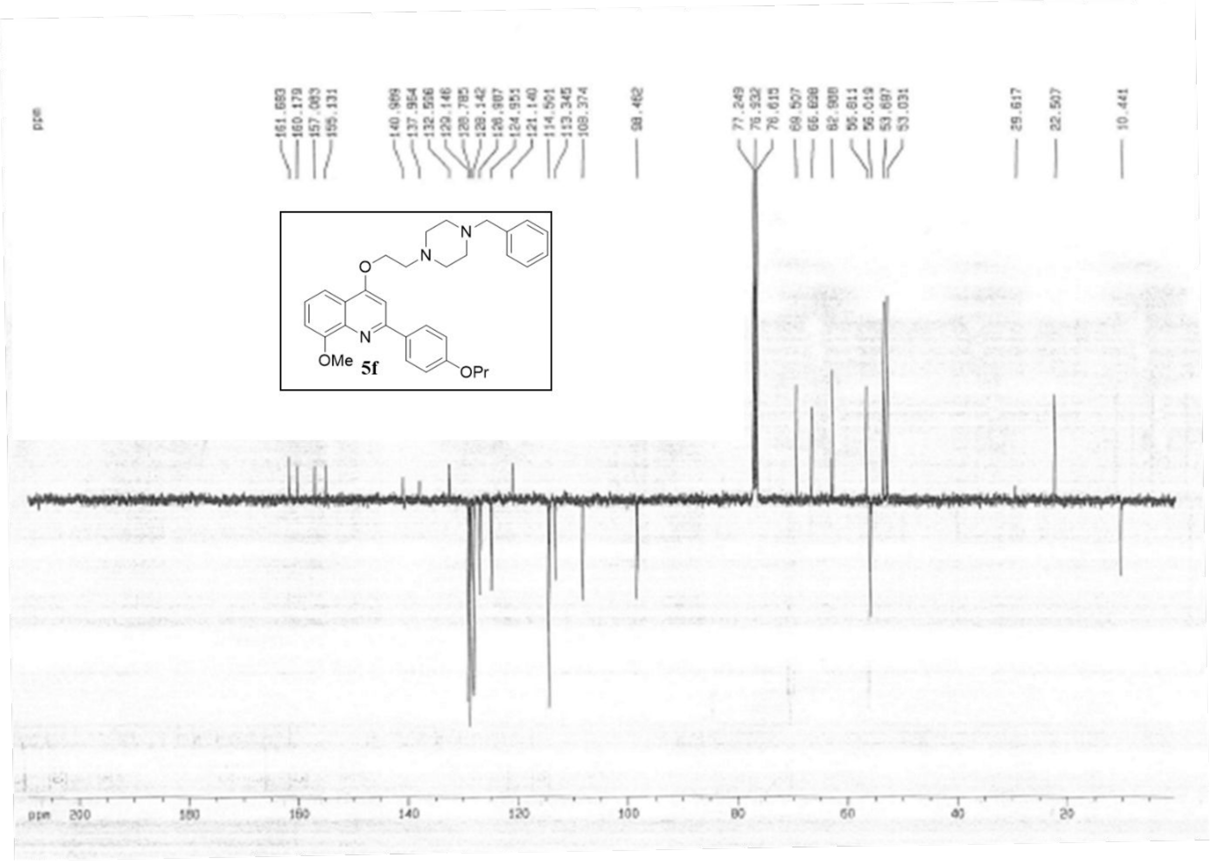

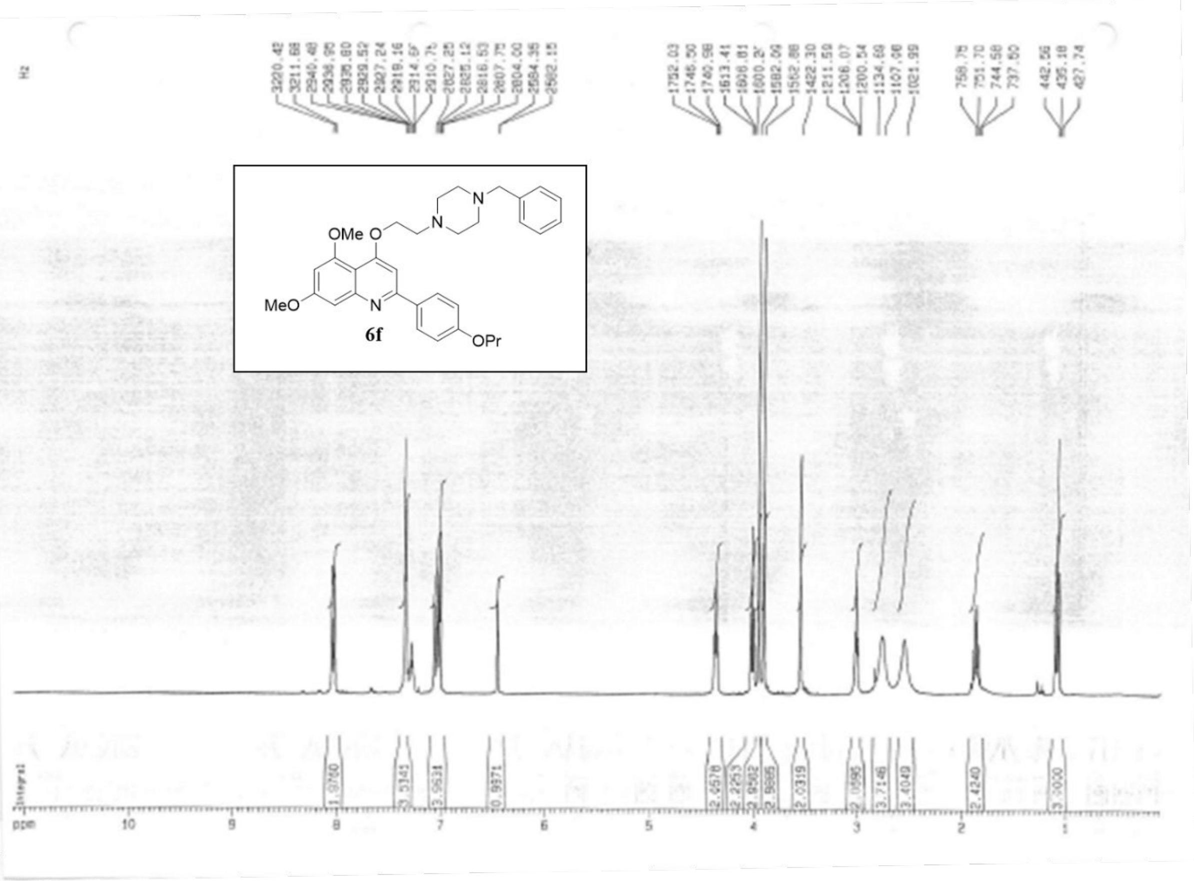
OMe
MeO
N
6f
OPr
ppm

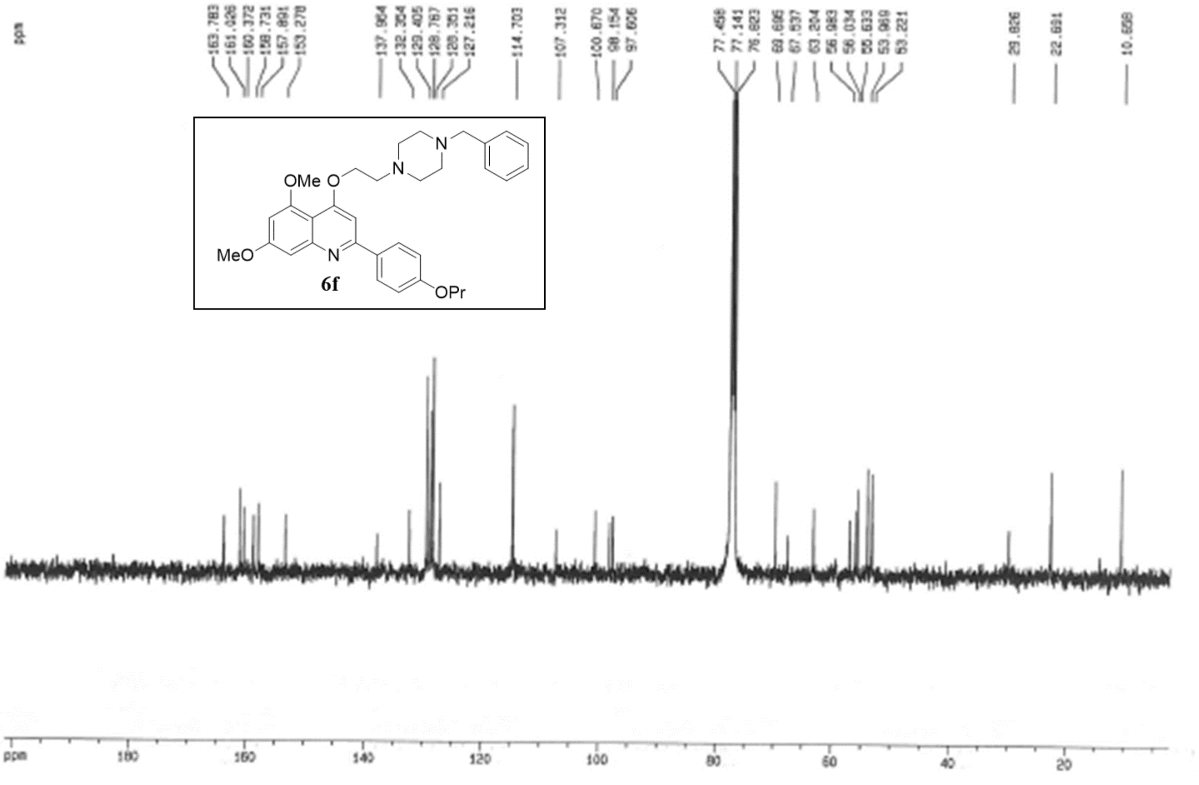
OMe
MeO
N
6f
OPr
ppm

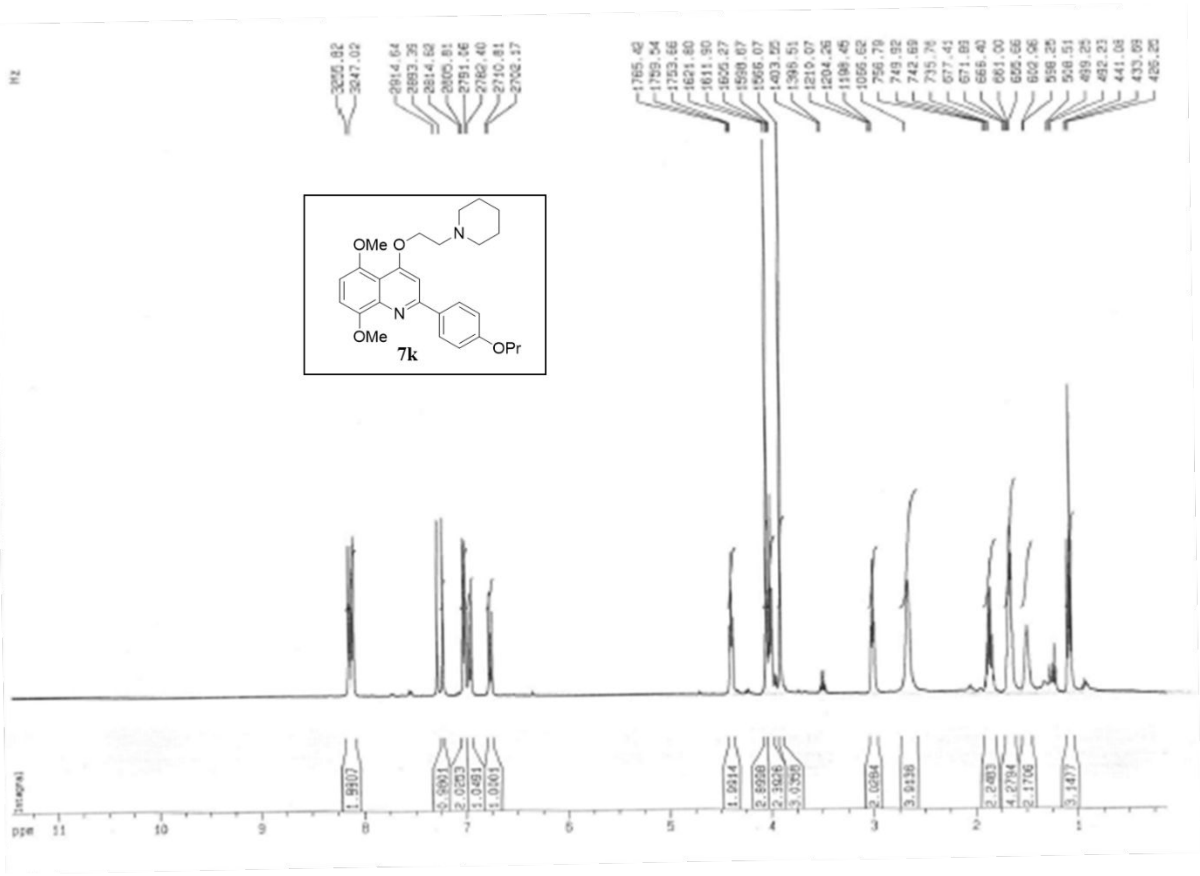
OMe
OMe
N
O
OPr
7k

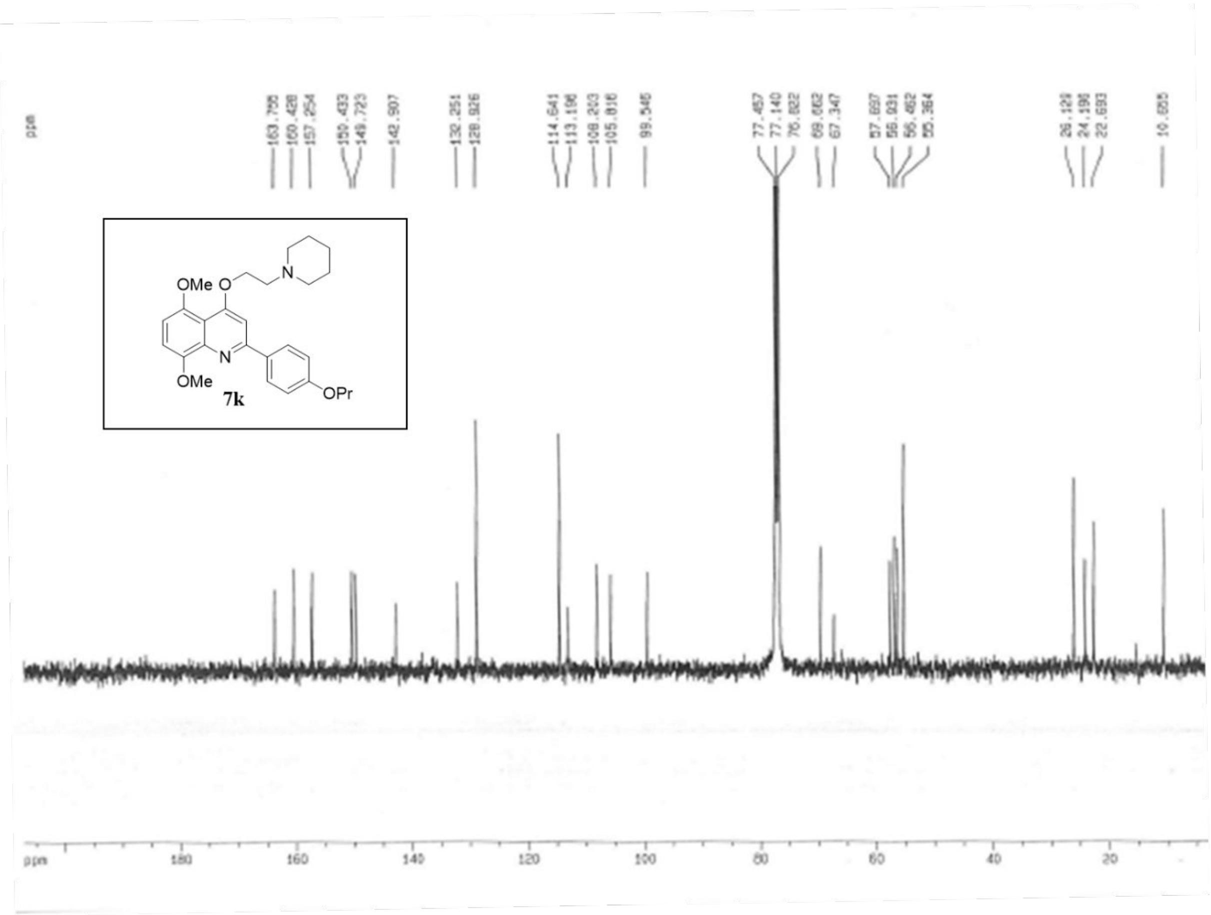

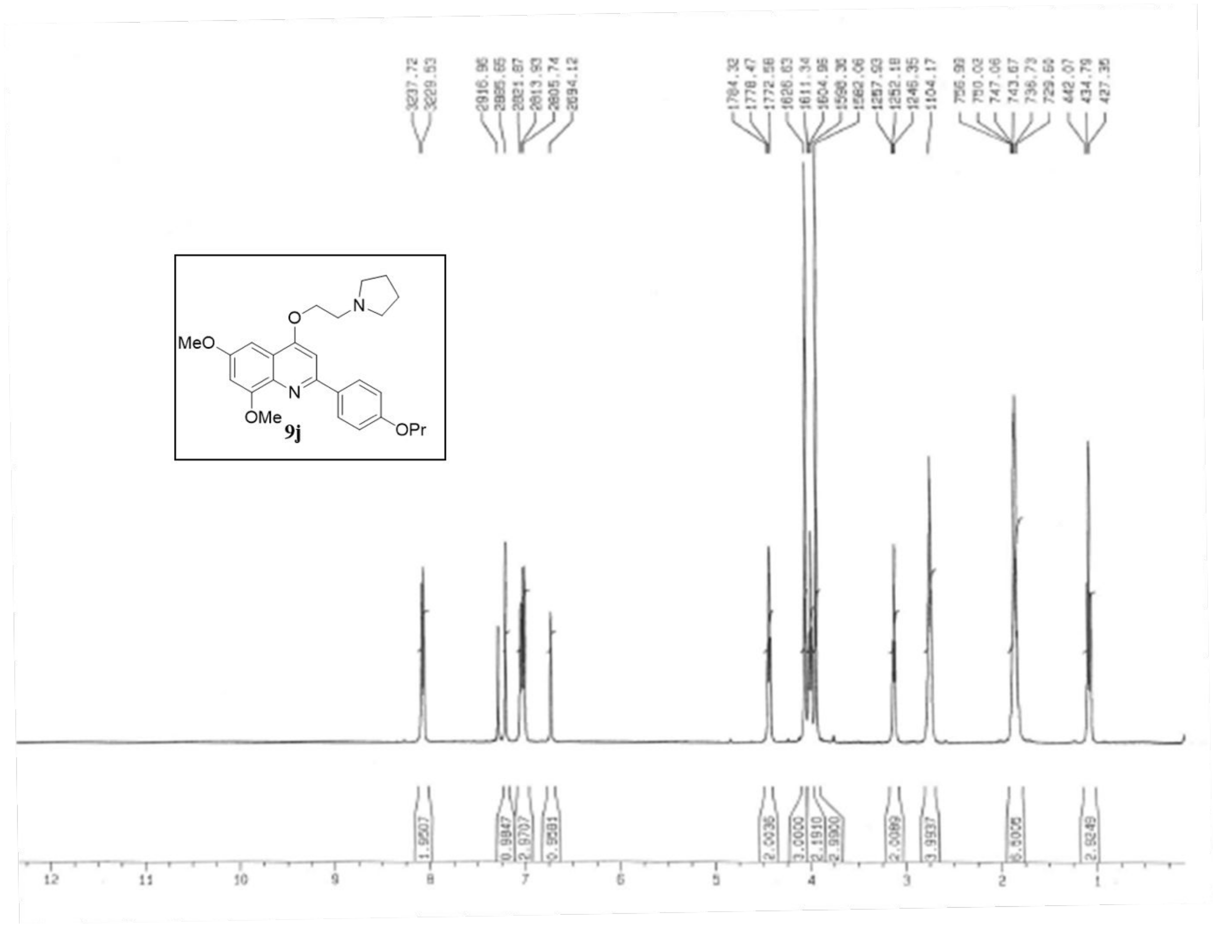

MeO
OMe
N
O
OPr
9j
12
11
10
9
8
7
6
5
4
3
2
1

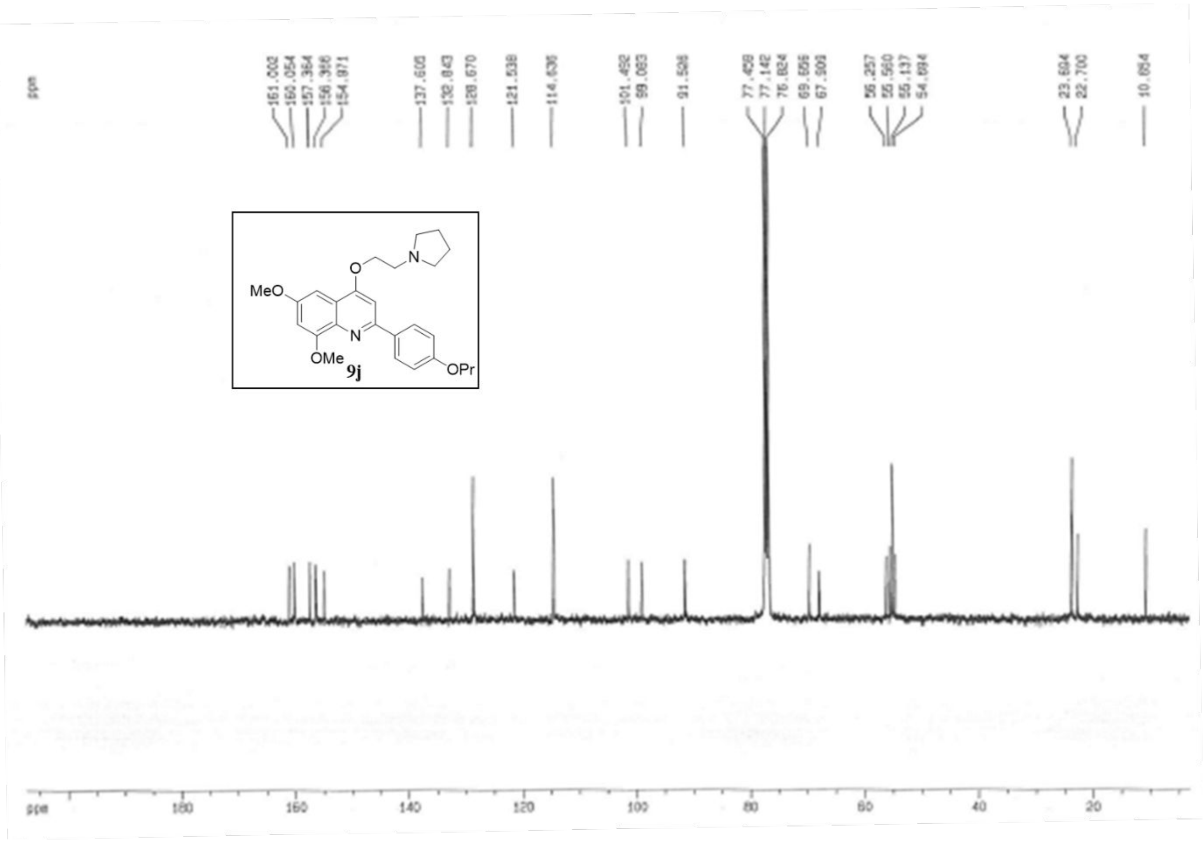

3.3 HPLC analysis of exemplary target compounds

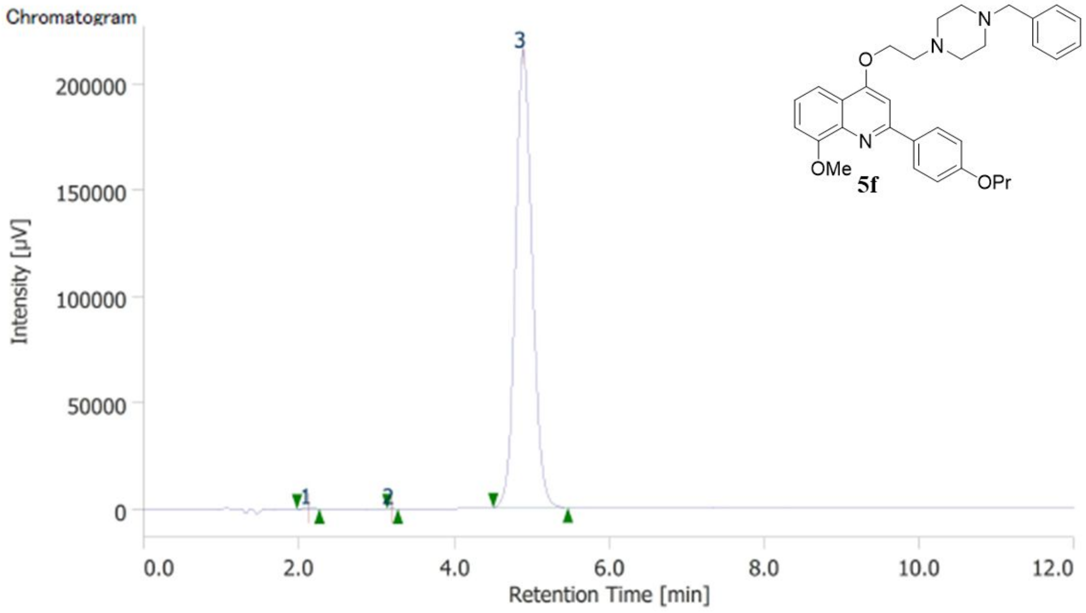

Peak Information

#	Peak Name	CH	tR [min]	Area [µV·sec]	Height [µV]	Area%	Height%	Quantity	Resolution
1	Unknown	5	2.140	3861	397	0.119	0.184	N/A	5.075
2	Unknown	5	3.203	703	147	0.022	0.068	N/A	6.401
3	Unknown	5	4.883	3242523	215192	99.859	99.748	N/A	N/A

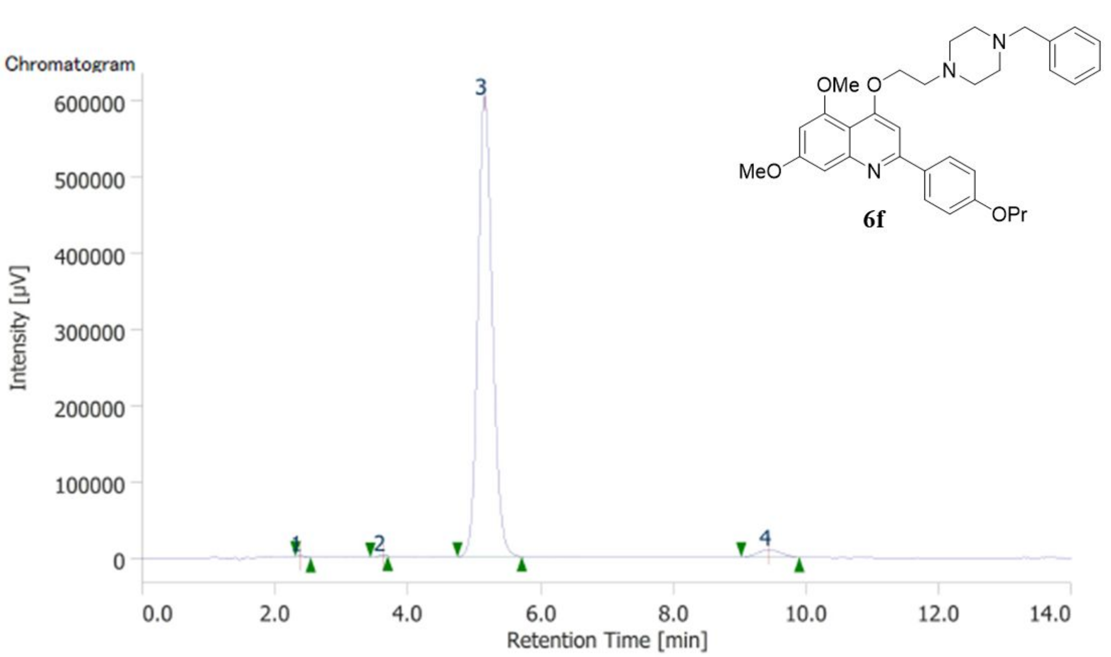

Peak Information

#	Peak Name	CH	tR [min]	Area [µV·sec]	Height [µV]	Area%	Height%	Quantity	Resolution
1	Unknown	5	2.377	9680	1354	0.103	0.219	N/A	6.262
2	Unknown	5	3.637	10884	1518	0.115	0.246	N/A	5.052
3	Unknown	5	5.140	9174102	604350	97.222	97.948	N/A	8.071
4	Unknown	5	9.433	241560	9790	2.560	1.587	N/A	N/A

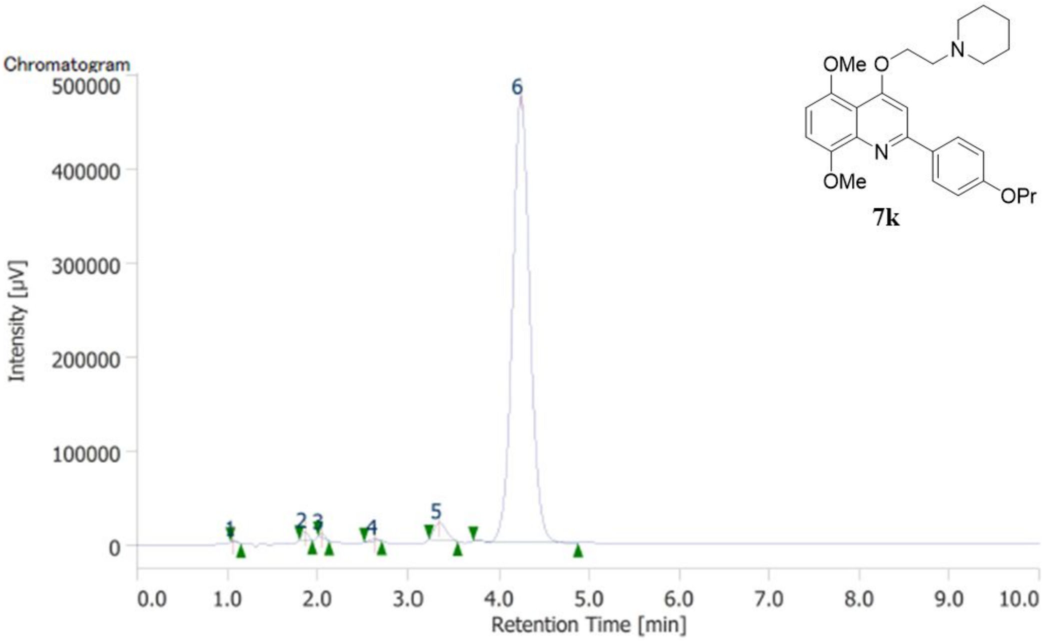

7k

Peak Information

#	Peak Name	CH	tR [min]	Area [µV·sec]	Height [µV]	Area%	Height%	Quantity	Resolution
1	Unknown	5	1.070	8537	2400	0.129	0.470	N/A	6.682
2	Unknown	5	1.863	40117	8267	0.605	1.621	N/A	1.369
3	Unknown	5	2.043	20084	4561	0.303	0.894	N/A	3.559
4	Unknown	5	2.643	16441	2241	0.248	0.439	N/A	2.960
5	Unknown	5	3.337	172785	18456	2.606	3.618	N/A	2.992
6	Unknown	5	4.237	6371482	474218	96.109	92.958	N/A	N/A

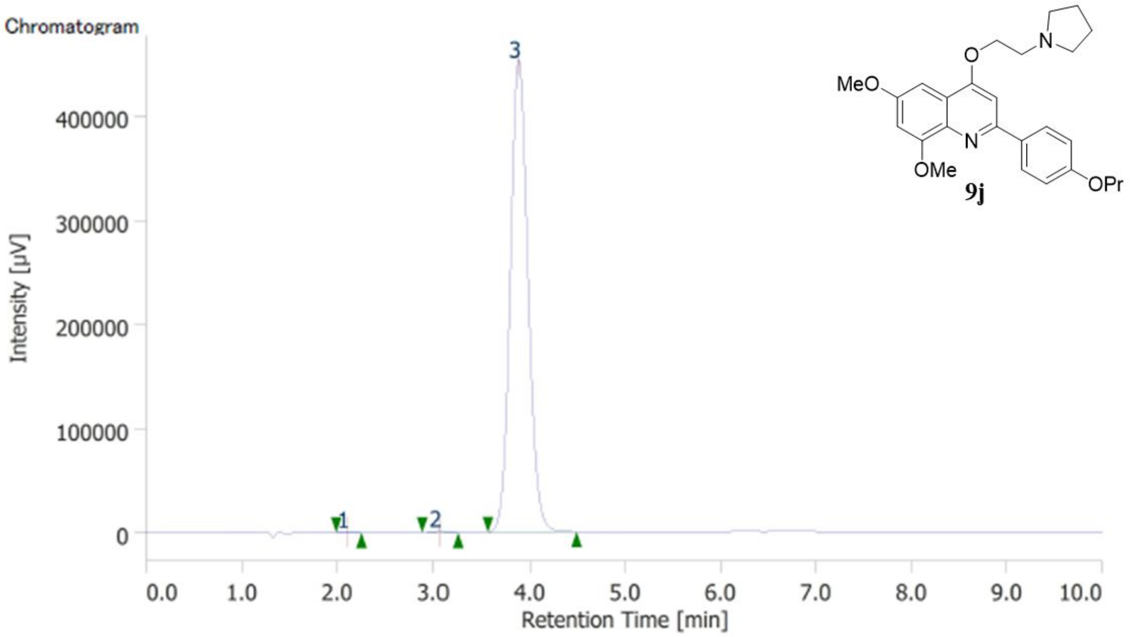

9j

Peak Information

#	Peak Name	CH	tR [min]	Area [µV·sec]	Height [µV]	Area%	Height%	Quantity	Resolution
1	Unknown	5	2.103	2454	334	0.042	0.073	N/A	3.815
2	Unknown	5	3.070	12170	1102	0.210	0.242	N/A	2.549
3	Unknown	5	3.883	5794180	453595	99.748	99.685	N/A	N/A

4 Biology

4.1 Cells and viruses

The SARS-CoV-2 isolate used in this study was the BetaCov/Belgium/GHB-03021/2020 (EPI ISL407976|2020-02-03), which was isolated from a Belgian patient returning from Wuhan in February 2020. The isolate was passaged 7 times on Vero E6 cells which introduced two series of amino acid deletions in the spike protein. [17] The infectious content of the virus stock was determined by titration on Vero E6 cells. Vero E6 cells were maintained in Dulbecco's modified Eagle's medium (DMEM; Gibco) supplemented with heat-inactivated 10% v/v fetal calf serum (FCS; Biowest) and 500 µg/mL Geneticin (Gibco) and kept under 5% CO_2 at 37°C. All SARS-CoV-2-related experimental work was performed in the certified, high-containment biosafety level-3 facilities of the Rega Institute at the KU Leuven (Belgium).

In the BSL2 facility, human embryonic lung fibroblasts (**HEL** 299; ATCC CCL-137) were cultured in Dulbecco's modified Eagle's medium (DMEM; Gibco catalog no. 41965-039) supplemented with 8% fetal bovine serum (FBS). The **HCoV-229E** (ATCC VR-740) and **HCoV-OC43** (ATCC VR-1558) stocks were obtained by inoculating a confluent monolayer of HEL 299 or HRT-18G cells, respectively. The supernatant was harvested after 3 days of incubation for HCoV-229E, or 7 days of incubation for HCoV-OC43, at 35 °C under 5% CO_2. After one freeze-thaw cycle and removal of cellular debris by centrifugation, aliquots were stored at −80 °C.

4.2 SARS-CoV-2 screening

The SARS-CoV-2 antiviral assay is derived from the previously established SARS-CoV assay.[18] In this assay, fluorescence of Vero E6-eGFP cells (provided by Dr. K. Andries J&JPRD; Beerse, Belgium) declines after infection with SARS-CoV-2 due to the cytopathogenic effect of the virus. In the presence of an antiviral compound, the cytopathogenicity is inhibited and the fluorescent signal rescued. Stock solutions of the various compounds in DMSO (10 mM) were prepared. On day -1, the test compounds were serially diluted in assay medium (DMEM supplemented with 2% v/v FCS). The plates were incubated (37 °C, 5% CO_2 and 95% relative humidity) overnight. On day 0, the diluted compounds were then mixed with SARS-CoV-2 at 20 TCID50/well and Vero E6-eGFP cells corresponding to a final density of 25,000 cells/well in 96-well blackview plates (Greiner Bio-One, Vilvoorde, Belgium). The plates were incubated in a humidified incubator at 37 °C and 5% CO_2. At 4 days p.i., the wells were examined for eGFP expression using an argon laser-scanning microscope. The microscope settings were excitation at 488 nm and emission at 510 nm and the fluorescence images of the wells were converted into signal values. The results were expressed as EC_{50} values

defined as the concentration of compound achieving 50% rescue of the virus-reduced eGFP signals as compared to the untreated virus-infected control cells. Toxicity of compounds in the absence of virus was evaluated in a standard MTS-assay as described previously.[19]

4.3 HCoV screening

For the antiviral evaluation of the 2-PhQs against HCoV-OC43 and HCoV-229E, HEL 299 cells were seeded into 384-well dishes at 5,000 cells per well and incubated overnight at 37 °C. Then, serial dilutions of the compounds were added to the cells prior to infection with HCoV-229E at 30 $TCID_{50}$ or with HCoV-OC43 at 50 $TCID_{50}$ per well. After 7 days incubation at 35 °C, the virus-induced cytopathogenic effect was measured colorimetrically by the formazan-based 3-(4,5-dimethylthiazol-2-yl)-5-(3-carboxymethoxyphenyl)-2-(4-sulfophenyl)-2H-tetrazolium (MTS) cell viability assay (CellTiter 96 AQueous One Solution Cell Proliferation Assay from Promega, Madison, WI), and the antiviral activity was expressed as the 50% effective concentration (EC_{50}). In parallel, the 50% cytotoxic concentration (CC_{50}) was derived from mock-infected cells.

4.4 Autophagy Inhibition assay

Immunofluorescence staining was performed according to standard procedures. Briefly, Vero E6 cells were seeded at a density of 20,000 cells per well in 8-well μ-slides (Ibidi). Cells were allowed to adhere overnight before compound treatment. After 4 hours of incubation, cells were fixed (4% paraformaldehyde in PBS), washed and permeabilized (0.2% Triton X-100 in PBS). Primary antibody used was rabbit anti-LC3B (L7543, Sigma) at a 1:200 dilution, and secondary antibody Alexa Fluor® 568 goat anti-rabbit (A11011, Invitrogen, ThermoFisher Scientific) was diluted 1:500. Cell nuclei were counterstained using 4’, 6-diamidino-2-phenylindole (DAPI) and the samples were imaged by confocal microscopy on a Leica TCS SP5 confocal microscope (Leica Microsystems), employing a HCX PL APO 63x (NA 1.2) water immersion objective. Different fluorochromes were detected sequentially using excitation lines of 405 nm (DAPI) and 568 nm (AlexaFluor 568) and emission was detected between 410 and 480 nm or between 566 and 670, respectively. Subsequently, fluorescence was read on a CellInsight CX5 High Content Reader (ThermoFisher Scientific). Cytoplasmic compartments were segmented and their average pixel intensities in the red channel were quantitated employing the HCS Studio Cell Analysis Software.

4.5 Determination of SARS-CoV-2 nsp13 unwinding-associated activity

SARS-CoV-2 nsp13, was kindly provided by professor Marcin Nowotny, Laboratory of Protein Structure, International Institute of Molecular and Cell Biology, Ks. Trojdena 4, Warsaw, 02-109, Poland.

A fluorescent-based helicase unwind assay was established using a double stranded DNA, with unpaired flap at the 5' termini. One strand of the duplex was labeled with Cy3, while the other contained a BHQ-2 quencher. An excess of a capture DNA oligo, with the same sequence as the one bearing the Cy3 but without the modification, was also added to the reaction mixture to prevent reannealing of the displaced strands.

The SARS-CoV-2 nsp13 unwinding-associated activity was measured in black 384 well plates (PerkinElmer), in 40 µl reaction volume containing 20 mM Tris–HCl, pH 7.2, 50 mM NaCl, 2 µM Hel Capture oligo (5'- TGG TGC TCG AAC AGT GAC -3') from Biomers, 5 mM $MgCl_2$, 5% DMSO or inhibitor and 1 nM of purified nsp13. The reaction mixture containing the enzyme was pre-incubated for 10 min with inhibitor at room temperature (RT). The reaction was started adding 1 mM ATP and 750 nM annealed DNA substrate (5'- AGT CTT CTC CTG GTG CTC GAA CAG TGA C-Cy3-3', 5'- BHQ-2-GTC ACT GTT CGA GCA CCA CCT CTT CTG A-3') from Biomers. After 15 min of incubation at 37 °C, products were measured with Victor Nivo (Perkin) at 530/580 nm. Experiments were performed in triplicate. Compound SSYA10-001[20] was used as positive control.

4.6 Determination of SARS-CoV-2 nsp13 ATPase-associated activity

The unwinding independent NTPase activity was determined by quantifying the green complex formed between the molybdate/malachite and the pyrophosphate released upon ATP hydrolysis.

The SARS-CoV-2 nsp13 ATPase helicase-associated activity was measured in a transparent 96 well plate (PerkinElmer), in 25 µl reaction volume containing 20 mM Tris–HCl, pH 7.2, 50 mM NaCl, 2 mM $MgCl_2$, 5% DMSO or inhibitor and 25 nM of purified nsp13. The reaction was started adding 400 µM ATP. After 30 min of incubation at 37 °C, 50 µl of Biomol® Green Reagent (Prod. No. BML-AK111, Enzo Lifescience) were added and reaction was incubated for 10 min at RT, protected from the light. Products were measured with Victor Nivo (Perkin) at 650 nm.

4.7 Determination of SARS-CoV-2 nsp12 RdRp activity

SARS-CoV-2 nsp12 was expressed from pET28a vector in BL21 DE3 cells, using LB medium. First step of purification consisted of affinity purification on Ni-Sepharose column, and the protein was eluted in a buffer contained 20 mM Tris pH8, 150 mM NaCl, 1M Imidazole and 4mM $MgCl_2$. The fraction contained the protein was loaded in a HiTrapQ-HP column and eluted in a buffer containing 20 mM Tris pH8, 1M NaCl and 4mM $MgCl_2$. The quality of the protein was analyzed through SDS-PAGE and the purified protein was stored at -80°C.The SARS-CoV-2 nsp12 RdRp activity was measured in a black 96 well plate (PerkinElmer), in 25 µl reaction volume containing 50 nM enzyme; 50 mM Tris HCl, 50 mM NaCl, 2.5 mM $MgCl_2$, 1 mM DTT, 10% glycerol, UTP 20 µM, RNase

inhibitor (Applied Biosystems™ N8080119) (20 units) pH 8.0, 0.625 μg/μl each well of PolyA and 0.03125 μg/ μl well of Oligo U. After 1 hour incubation at 37°C, 200 rpm 170 μl of PicoGreen working solution (EnzCheck RT kit E-22064) diluted 345 folds in 1X Tris EDTA buffer (10 mM TrisHCl, 1 mM EDTA, pH 7.5) were added. Products were measured with Victor Nivo (Perkin) at 320/480 nm. Compound F243[21] was used as positive control.

5. References

(1) Corona, A.; Desantis, J.; Massari, S.; Distinto, S.; Masaoka, T.; Sabatini, S.; Esposito, F.; Manfroni, G.; Maccioni, E.; Cecchetti, V.; Pannecouque, C.; Le Grice, S. F. J.; Tramontano, E.; Tabarrini, O. Studies on Cycloheptathiophene-3-Carboxamide Derivatives as Allosteric HIV-1 Ribonuclease H Inhibitors. *ChemMedChem* **2016**, *11* (16), 1709–1720. https://doi.org/10.1002/CMDC.201600015.

(2) Desantis, J.; Nannetti, G.; Massari, S.; Barreca, M. L.; Manfroni, G.; Cecchetti, V.; Palù, G.; Goracci, L.; Loregian, A.; Tabarrini, O. Exploring the Cycloheptathiophene-3-Carboxamide Scaffold to Disrupt the Interactions of the Influenza Polymerase Subunits and Obtain Potent Anti-Influenza Activity. *Eur. J. Med. Chem.* **2017**, *138*, 128–139. https://doi.org/10.1016/j.ejmech.2017.06.015.

(3) Pismataro, M. C.; Felicetti, T.; Bertagnin, C.; Nizi, M. G.; Bonomini, A.; Barreca, M. L.; Cecchetti, V.; Jochmans, D.; De Jonghe, S.; Neyts, J.; Loregian, A.; Tabarrini, O.; Massari, S. 1,2,4-Triazolo[1,5-a]Pyrimidines: Efficient One-Step Synthesis and Functionalization as Influenza Polymerase PA-PB1 Interaction Disruptors. *Eur. J. Med. Chem.* **2021**, *221*, 113494–113511. https://doi.org/10.1016/j.ejmech.2021.113494.

(4) Nizi, M. G.; Desantis, J.; Nakatani, Y.; Massari, S.; Mazzarella, M. A.; Shetye, G.; Sabatini, S.; Barreca, M. L.; Manfroni, G.; Felicetti, T.; Rushton-Green, R.; Hards, K.; Latacz, G.; Satała, G.; Bojarski, A. J.; Cecchetti, V.; Kolář, M. H.; Handzlik, J.; Cook, G. M.; Franzblau, S. G.; Tabarrini, O. Antitubercular Polyhalogenated Phenothiazines and Phenoselenazine with Reduced Binding to CNS Receptors. *Eur. J. Med. Chem.* **2020**, *201*, 112420–112433. https://doi.org/10.1016/j.ejmech.2020.112420.

(5) Venkannagari, H.; Verheugd, P.; Koivunen, J.; Haikarainen, T.; Obaji, E.; Ashok, Y.; Narwal, M.; Pihlajaniemi, T.; Lüscher, B.; Lehtiö, L. Small-Molecule Chemical Probe Rescues Cells from Mono-ADP-Ribosyltransferase ARTD10/PARP10-Induced Apoptosis and Sensitizes Cancer Cells to DNA Damage. *Cell Chem. Biol.* **2016**, *23* (10), 1251–1260. https://doi.org/10.1016/j.chembiol.2016.08.012.

(6) Sancineto, L.; Iraci, N.; Massari, S.; Attanasio, V.; Corazza, G.; Barreca, M. L.; Sabatini, S.; Manfroni, G.; Avanzi, N. R.; Cecchetti, V.; Pannecouque, C.; Marcello, A.; Tabarrini, O. Computer-Aided Design, Synthesis and Validation of 2-Phenylquinazolinone Fragments as CDK9 Inhibitors with Anti-HIV-1 Tat-Mediated Transcription Activity. *ChemMedChem* **2013**, *8*, 1941–1953. https://doi.org/10.1002/cmdc.201300287.

(7) Tabarrini, O.; Cecchetti, V.; Fravolini, A.; Nocentini, G.; Barzi, A.; Sabatini, S.; Miao, H.; Sissi, C. Design and Synthesis of Modified Quinolones as Antitumoral Acridones. *J. Med.*

Chem. **1999**, *42*, 2136–2144. https://doi.org/10.1021/jm980324m.

(8) Cecchetti, V.; Parolin, C.; Moro, S.; Pecere, T.; Filipponi, E.; Calistri, A.; Tabarrini, O.; Gatto, B.; Palumbo, M.; Fravolini, A.; Palu, G. 6-Aminoquinolones as New Potential Anti-HIV Agents. *J. Med. Chem.* **2000**, *43* (20), 3799–3802. https://doi.org/10.1021/jm9903390.

(9) Donalisio, M.; Massari, S.; Argenziano, M.; Manfroni, G.; Cagno, V.; Civra, A.; Sabatini, S.; Cecchetti, V.; Loregian, A.; Cavalli, R.; Lembo, D.; Tabarrini, O. Ethyl 1,8-Naphthyridone-3-Carboxylates Downregulate Human Papillomavirus-16 E6 and E7 Oncogene Expression. *J. Med. Chem.* **2014**, *57* (13), 5649–5663. https://doi.org/10.1021/JM500340H.

(10) Matthes, F.; Massari, S.; Bochicchio, A.; Schorpp, K.; Schilling, J.; Weber, S.; Offermann, N.; Desantis, J.; Wanker, E.; Carloni, P.; Hadian, K.; Tabarrini, O.; Rossetti, G.; Krauss, S. Reducing Mutant Huntingtin Protein Expression in Living Cells by a Newly Identified RNA CAG Binder. *ACS Chem. Neurosci.* **2018**, *9* (6), 1399–1408. https://doi.org/10.1021/ACSCHEMNEURO.8B00027/SUPPL_FILE/CN8B00027_SI_001.PDF.

(11) Gatto, B.; Tabarrini, O.; Massari, S.; Giaretta, G.; Sabatini, S.; Del Vecchio, C.; Parolin, C.; Fravolini, A.; Palumbo, M.; Cecchetti, V. 2-Phenylquinolones as Inhibitors of the HIV-1 Tat-TAR Interaction. *ChemMedChem* **2009**, *4* (6), 935–938. https://doi.org/10.1002/cmdc.200800437.

(12) Sabatini, S.; Gosetto, F.; Manfroni, G.; Tabarrini, O.; Kaatz, G. W.; Patel, D.; Cecchetti, V. Evolution from a Natural Flavones Nucleus to Obtain 2-(4-Propoxyphenyl)Quinoline Derivatives As Potent Inhibitors of the S.Aureus NorA Efflux Pump. *J. Med. Chem* **2011**, *54*, 5722–5736. https://doi.org/10.1021/jm200370y.

(13) Sabatini, S.; Gosetto, F.; Iraci, N.; Barreca, M. L.; Massari, S.; Sancineto, L.; Manfroni, G.; Tabarrini, O.; Dimovska, M.; Kaatz, G. W.; Cecchetti, V. Re-Evolution of the 2-Phenylquinolines: Ligand-Based Design, Synthesis, and Biological Evaluation of a Potent New Class of Staphylococcus Aureus NorA Efflux Pump Inhibitors to Combat Antimicrobial Resistance. *J. Med. Chem.* **2013**, *56*, 4975–4989. https://doi.org/10.1021/jm400262a.

(14) Felicetti, T.; Cannalire, R.; Pietrella, D.; Latacz, G.; Lubelska, A.; Manfroni, G.; Barreca, M. L.; Massari, S.; Tabarrini, O.; Kieć-Kononowicz, K.; Schindler, B. D.; Kaatz, G. W.; Cecchetti, V.; Sabatini, S. 2-Phenylquinoline S. Aureus NorA Efflux Pump Inhibitors: Evaluation of the Importance of Methoxy Group Introduction. *J. Med. Chem.* **2018**, *61* (17), 7827–7848. https://doi.org/10.1021/acs.jmedchem.8b00791.

(15) Felicetti, T.; Mangiaterra, G.; Cannalire, R.; Cedraro, N.; Pietrella, D.; Astolfi, A.; Massari, S.; Tabarrini, O.; Manfroni, G.; Barreca, M. L.; Cecchetti, V.; Biavasco, F.; Sabatini, S. C-2 Phenyl Replacements to Obtain Potent Quinoline-Based Staphylococcus Aureus NorA Inhibitors. *J. Enzyme Inhib. Med. Chem.* **2020**, *35* (1), 584–597. https://doi.org/10.1080/14756366.2020.1719083.

(16) Cannalire, R.; Mangiaterra, G.; Felicetti, T.; Astolfi, A.; Cedraro, N.; Massari, S.; Manfroni, G.; Tabarrini, O.; Vaiasicca, S.; Barreca, M. L.; Cecchetti, V.; Biavasco, F.; Sabatini, S. Structural Modifications of the Quinolin-4-Yloxy Core to Obtain New Staphylococcus Aureus NorA Inhibitors. *Int. J. Mol. Sci.* **2020**, *21* (19), 1–18. https://doi.org/10.3390/ijms21197037.

(17) Boudewijns, R.; Thibaut, H. J.; Kaptein, S. J. F.; Li, R.; Vergote, V.; Seldeslachts, L.; Van Weyenbergh, J.; De Keyzer, C.; Bervoets, L.; Sharma, S.; Liesenborghs, L.; Ma, J.; Jansen, S.; Van Looveren, D.; Vercruysse, T.; Wang, X.; Jochmans, D.; Martens, E.; Roose, K.; De

Vlieger, D.; Schepens, B.; Van Buyten, T.; Jacobs, S.; Liu, Y.; Martí-Carreras, J.; Vanmechelen, B.; Wawina-Bokalanga, T.; Delang, L.; Rocha-Pereira, J.; Coelmont, L.; Chiu, W.; Leyssen, P.; Heylen, E.; Schols, D.; Wang, L.; Close, L.; Matthijnssens, J.; Van Ranst, M.; Compernolle, V.; Schramm, G.; Van Laere, K.; Saelens, X.; Callewaert, N.; Opdenakker, G.; Maes, P.; Weynand, B.; Cawthorne, C.; Vande Velde, G.; Wang, Z.; Neyts, J.; Dallmeier, K. STAT2 Signaling Restricts Viral Dissemination but Drives Severe Pneumonia in SARS-CoV-2 Infected Hamsters. *Nat. Commun.* **2020**, *11* (1), 5838–5848. https://doi.org/10.1038/s41467-020-19684-y.

(18) Ivens, T.; Van Den Eynde, C.; Van Acker, K.; Nijs, E.; Dams, G.; Bettens, E.; Ohagen, A.; Pauwels, R.; Hertogs, K. Development of a Homogeneous Screening Assay for Automated Detection of Antiviral Agents Active against Severe Acute Respiratory Syndrome-Associated Coronavirus. *J. Virol. Methods* **2005**, *129*, 56–63. https://doi.org/10.1016/j.jviromet.2005.05.010.

(19) Jochmans, D.; Leyssen, P.; Neyts, J. A Novel Method for High-Throughput Screening to Quantify Antiviral Activity against Viruses That Induce Limited CPE. *J. Virol. Methods* **2012**, *183* (2), 176–179. https://doi.org/10.1016/j.jviromet.2012.04.011.

(20) Adedeji, A. O.; Singh, K.; Calcaterra, N. E.; DeDiego, M. L.; Enjuanes, L.; Weiss, S.; Sarafianos, S. G. Severe Acute Respiratory Syndrome Coronavirus Replication Inhibitor That Interferes with the Nucleic Acid Unwinding of the Viral Helicase. *Antimicrob. Agents Chemother.* **2012**, *56* (9), 4718–4728. https://doi.org/https://doi.org/10.1128/AAC.00957-12.

(21) Dejmek, M.; Konkol'ová, E.; Eyer, L.; Straková, P.; Svoboda, P.; Šála, M.; Krejčová, K.; Růžek, D.; Boura, E.; Nencka, R. Non-Nucleotide RNA-Dependent RNA Polymerase Inhibitor That Blocks SARS-CoV-2 Replication. *Viruses* **2021**, *13* (8), 1585. https://doi.org/10.3390/V13081585.
